# Notes on four species of *Russula* subgenus *Heterophyllidiae* (Russulaceae, Russulales) from southern China

**DOI:** 10.3389/fmicb.2023.1140127

**Published:** 2023-03-21

**Authors:** Yun-Xiao Han, Zhi-Qun Liang, Nian-Kai Zeng

**Affiliations:** ^1^Key Laboratory of Tropical Translational Medicine of Ministry of Education, School of Pharmacy, Hainan Medical University, Haikou, China; ^2^College of Science, Hainan University, Haikou, China

**Keywords:** ectomycorrhizal fungi, molecular phylogeny, morphology, new taxa, taxonomy

## Abstract

*Heterophyllidiae*, one of the main subgenus of *Russula* (Russulaceae, Russulales), is both ecologically and economically important. Although many studies have focused on subgenus *Heterophyllidiae* in China, the diversity, taxonomy, and molecular phylogeny still remained incompletely understood. In the present study, two new species, *R. discoidea* and *R. niveopicta*, and two known taxa, *R. xanthovirens* and *R. subatropurpurea,* were described based on morphology and molecular phylogenetic analyses of ITS and 28S DNA sequences with new collections of subgenus *Heterophyllidiae* from southern China. Both morphological and phylogenetic analyses consistently confirmed that *R. niveopicta* and *R. xanthovirens* belong to the subsect. *Virescentinae*, *R. discoidea* and *R. subatropurpurea* come under subsect. *Heterophyllae*, and *R. prasina* is synonymized with *R. xanthovirens*.

## Introduction

The genus *Russula* Pers. was established by Persoon (1796). Recently, the genus has been divided into eight subgenera: *Archaeae* Buyck and V. Hofst., *Brevipedum* Buyck and V. Hofst., *Compactae* (Fr.) Bon, *Crassotunicatae* Buyck and V. Hofst., *Glutinosae* Buyck and X. H. Wang, *Heterophyllidiae* Romagn., *Malodorae* Buyck and V. Hofst., and *Russula* Pers. (Buyck et al., 2018, 2020). Among them, subg. *Heterophyllidiae* is characterized by medium to large basidiomata, adnate lamellae, rare or no lamellulae, a mild to strongly acrid taste, white or cream spore prints, an inamyloid or partly amyloid suprahilar spot on the spores, absence of primordial hyphae, a suprapellis comprising mainly inflated hyphal extremities, and mycorrhizal properties (Knudsen and Borgen, 1982; Romagnesi, 1987; Buyck et al., 1996, 2018), which has received much attention. The subgenus includes six sections: *Aureotactineae* R. Heim, *Heterophyllae* Fr., *Ilicinae* Romagn., *Indolentinae* Melzer and Zvára, *Ingratae* Quel., and *Virescentinae* (Singer) Sarnari, and two subsections: *Cyanoxanthinae* Singer and *Substriatinae* X. H. Wang and Buyck (Persoon, 1796; Buyck et al., 2018, 2020).

In previous studies, about 161 species within subg. *Heterophyllidiae* were revealed around the world (Ying, 1983; Li et al., 2013, 2015, 2018, 2019, 2021; Chen et al., 2014, 2019, 2021a,b,c,d; Zhao et al., 2015; Zhang et al., 2017; Li and Deng, 2018; Song et al., 2018a,b, 2020; Wang et al., 2019; Yuan et al., 2019; Ghosh et al., 2020; Wisitrassameewong et al., 2020, 2022; Vera et al., 2021; Altaf et al., 2022; Han et al., 2022; Song, 2022). Moreover, the edibility and poisonousness of the subgenus have also been noted, e.g., edible species, *R. maguanensis* J. Wang, X. H. Wang, Buyck and T. Bau, *R. substriata* J. Wang, X. H. Wang, Buyck and T. Bau, *R. vesca* Fr., and *R. viridirubrolimbata* J. Z. Ying; and poisonous mushroom *R. senecis* S. Imai (Mao, 2006; Chen et al., 2014; Tolgor et al., 2014; Wang, 2019; Wu et al., 2019).

In China, 38 species of subg. *Heterophyllidiae* have also been described/reported, which greatly enriched the species diversity of this subgenus (Ying, 1983; Chou and Wang, 2005; Li et al., 2013, 2015, 2018, 2019, 2021; Chen et al., 2014, 2019, 2021a,b,c,d; Zhao et al., 2015; Zhang et al., 2017; Li and Deng, 2018; Song et al., 2018a,b, 2020; Wang et al., 2019; Yuan et al., 2019; Han et al., 2022; Song, 2022). Even so, the diversity and taxonomy still remained incompletely understood in the country. In the present study, with new collections of subg. *Heterophyllidiae* made from southern China, two new species were described, and the information of two known taxa was updated based on the morphological and molecular phylogenetic analyses, aiming to contribute to the knowledge of this subgenus.

## Materials and methods

### Morphological studies

Specimens were photographed under daylight in the field, and their macroscopic characteristics were measured and recorded based on fresh basidiomata. Specimens were dried at 50°C–60°C and then deposited in the Fungal Herbarium of Hainan Medical University (FHMU) (Index Herbariorum), Haikou City, Hainan Province, China. Color codes follow Kornerup and Wanscher (1981). The description templates and terminology of the micromorphological characters referred to Adamčík et al. (2019). The pileipellis section taken from the pileus between the center and margin, and the stipitipellis from the middle part along the longitudinal axis of the stipe were also observed (Zeng et al., 2013). Estimates of spore ornamentation density from scanning electron microscopy pictures follow Adamčík and Marhold (2000). The hymenial cystidia density estimates refer to Buyck (1991). The pileipellis ortho- or metachromatic reactions were examined in Cresyl Blue after Buyck (1989). Sulfovanillin (SV) was used to observe color changes in cystidia contents (Caboň et al., 2017). Observations and measurements of microscopic features were made in 1% Congo Red, 5% potassium hydroxide (KOH), or Melzer’s reagent. The size of the basidiospore was measured with the exclusion of ornamentation and apiculus. The basidiospores were examined using a TM4000Plus or Zeiss Sigma 300 scanning electron microscope (SEM). All the microscopic structures were drawn by free hand. The number of measured basidiospores is given as n/m/p, where “n” represents the total number of basidiospores measured from “m” basidiomata of “p” collections. Dimensions of basidiospores are presented as (a–)b–e–c(−d), where the range “b–c” represents a minimum of 90% of the measured values (5th to 95th percentile), and extreme values (a and d), whenever present (a < 5th percentile, d > 95th percentile), are in parentheses, and “e” refers to the average length/width of basidiospores. “Q” refers to the length/width ratio of basidiospores; “Qm” refers to the average “Q” of basidiospores and is given with standard deviation.

### Molecular procedures

Total genomic DNA was extracted from collections dried with silica gel using the Plant Genomic DNA Kit (CWBIO, Beijing, China) according to the manufacturer’s instructions. Primer pairs used for amplification were as follows: nuc 28S rDNA D1-D2 domains (28S) with LR0R/LR5 (Vilgalys and Hester, 1990; James et al., 2006), nuc rDNA region encompassing the internal transcribed spacers 1 and 2, along with the 5.8S rDNA (ITS) with ITS5/ITS4 (White et al., 1990), and EF1-F/EF1-R (Mikheyev et al., 2006) were used for the translation elongation factor 1-α gene (*TEF1*). PCR reactions were performed for 4 min of initial denaturation at 95°C, followed by 35 cycles of denaturation at 94°C for 30 s, annealing at the appropriate temperature (52°C for 28S and ITS; 53°C for *TEF1*) for 30 s, extension at 72°C for 120 s, and a final extension at 72°C for 7 min. Amplified PCR products were purified using the DNA Purification Kit (TIANGEN, Beijing, China) according to the manufacturer’s instructions and then directly sequenced using a BigDye terminator v3.1 kit and an ABI 3730xl DNA Analyzer (Guangzhou Branch of BGI, China) with the same primers used for PCR amplification. DNA sequences were compiled with BioEdit v7.0.9 (Hall, 1999) and then deposited in GenBank (Table 1).

**Table 1:** Taxa information and GenBank accession numbers of DNA sequences used in this study.

Taxa	Voucher	Locality	GenBank accession Nos.		Reference
			ITS	28S	
*Russula aeruginea*	AT2003017	Sweden	DQ421999	—	Buyck et al. (2008)
*Russula* aff*. crustosa*	BB 06.616	Canada	—	KU237461	Buyck et al. (2018)
*Russula* aff. *virescens*	BB 09.021	New Caledonia	—	KU237582	Buyck et al. (2018)
*Russula albidogrisea*	K15091234	Guangdong, southern China	KY767807	—	Das et al. (2017)
*Russula albidogrisea*	RITF1871	China	MW397095	MW397128	Unpublished
*Russula albolutea*	RITF2653	Hubei, central China	MT672478	MW397120	Chen et al. (2021b)
*Russula albolutea*	RITF4460	Chongqing, southwestern China	—	MW397121	Chen et al. (2021b)
*Russula albolutea*	RITF4461	Yunnan, southwestern China	—	MW397122	Chen et al. (2021b)
*Russula albolutea*	RITF4462	Yunnan, southwestern China	—	MW397123	Chen et al. (2021b)
*Russula amoena*	SAV F–3147	Slovakia	MT017544	—	Wisitrassameewong et al. (2020)
*Russula aureoviridis*	H16082612	Guangdong, southern China	KY767809	—	Das et al. (2017)
*Russula aureoviridis*	RITF4709	Guangdong, southern China	MW646980	MW646992	Chen et al. (2021a)
*Russula bella*	SFC20170819-05	South Korea	MT017552	—	Wisitrassameewong et al. (2020)
*Russula bubalina*	K15052614	Guangdong, southern China	MG018742	—	Li et al. (2019)
*Russula bubalina*	RITF1863	China	MW397097	—	Unpublished
*Russula* cf. *crustosa*	DSL002	Thailand	MT559557	—	Kaewgrajang et al. (2020)
*Russula* cf. *pseudobubalina*	HKAS122431	Yunnan, southwestern China	ON794290	—	Wang et al. (2022)
*Russula* cf. *vesca*	BB 06.525	Mexico	—	KU237465	Buyck et al. (2018)
*Russula crustosa*	BPL265	United States	KT933966	KT933826	Looney et al. (2016)
*“Russula crustosa”*	MHHNU 7960	China	OM760651	—	Unpublished
*Russula cyanoxantha*	FH 12–201	Germany	KR364093	KR364225	De Crop et al. (2017)
*Russula cyanoxantha*	RITF4682	Guangdong, southern China	MW646981	MW646993	Chen et al. (2021a)
*Russula cyanoxantha*	UE29.09.2002–2	France	DQ422033	—	Buyck et al. (2008)
*Russula dinghuensis*	GDGM45244	Guangdong, southern China	KU863579	—	Zhang et al. (2017)
*Russula dinghuensis*	RITF5142	China	MW646982	MW646994	Chen et al. (2021a)
***Russula discoidea***	**N.K. Zeng4895 (FHMU4847)**	**Hainan, southern China**	**OP837469**	**OP837459**	**Present study**
***Russula discoidea***	**N.K. Zeng4968 (FHMU5535)**	**Hainan, southern China**	**—**	**OP837460**	**Present study**
*Russula grisea*	UE2005.08.16–01	Sweden	DQ422030	—	Buyck et al. (2008)
*Russula grisea*	FH12234	Germany	KT934006	KT933867	Looney et al. (2016)
*Russula grisea*	BB 07.184	Slovakia	—	KU237509	Buyck et al. (2018)
*Russula heterophylla*	UE20.08.2004–2	Sweden	DQ422006	—	Buyck et al. (2008)
*Russula ilicis*	563IC52	Europe	AY061682	—	Miller and Buyck (2002)
*Russula ilicis*	MF 00.300	Italy	—	KU237595	Buyck et al. (2018)
*Russula ionochlora*	BB 07.338	Slovakia	—	KU237508	Buyck et al. (2018)
*Russula lakhanpalii*	AG 17–1,584	India	MN262088	—	Ghosh et al. (2020)
*Russula lakhanpalii*	RITF2600	China	MW646983	MW646995	Chen et al. (2021a)
*Russula langei*	BB 07.792	France	—	KU237510	Buyck et al. (2018)
*Russula lotus*	RITF499	China	MK860699	MW397129	Song et al. (2019)
*Russula luofuensis*	RITF4706	Guangdong, southern China	MW646973	MW646985	Chen et al. (2021a)
*Russula luofuensis*	RITF4707	Guangdong, southern China	MW646974	MW646986	Chen et al. (2021a)
*Russula luofuensis*	RITF4708	Guangdong, southern China	MW646975	MW646987	Chen et al. (2021a)
*Russula luofuensis*	RITF4712	Guangdong, southern China	MW646976	MW646988	Chen et al. (2021a)
*Russula luofuensis*	RITF4714	Guangdong, southern China	MW646977	MW646989	Chen et al. (2021a)
*Russula maguanensis*	XHW4765	Yunnan, southwestern China	MH724918	MH714537	Wang et al. (2019)
*Russula mariae*	HCCN19111	South Korea	KF361762	KF361812	Park et al. (2013)
*Russula mariae*	BB 07.038	United States	—	KU237538	Buyck et al. (2018)
*Russula medullata*	BB 07.252	Slovakia	—	KU237546	Buyck et al. (2018)
*Russula mustelina*	FH12226	Germany	KT934005	KT933866	Looney et al. (2016)
*Russula mustelina*	SA 09.88	Slovakia	—	KU237596	Buyck et al. (2018)
***Russula niveopicta***	**N.K. Zeng1413 (FHMU958)**	**Fujian, southeastern China**	**OP837461**	**OP837453**	**Present study**
***Russula niveopicta***	**N.K. Zeng1395 (FHMU941)**	**Fujian, southeastern China**	**OP837462**	**OP837454**	**Present study**
***Russula niveopicta***	**N.K. Zeng2252 (FHMU1497)**	**Hainan, southern China**	**OP837463**	**OP837455**	**Present study**
***Russula niveopicta***	**N.K. Zeng1408 (FHMU953)**	**Fujian, southeastern China**	**OP837464**	**OP837456**	**Present study**
*Russula orientipurpurea*	SFC20170819-08	South Korea	MT017550	—	Wisitrassameewong et al. (2020)
*Russula orientipurpurea*	SFC20170725-37	South Korea	MT017548	—	Wisitrassameewong et al. (2020)
*Russula pallidula*	RITF2613	Zhejiang, eastern China	MH027958	MH027960	Chen et al. (2019, 2021a)
*Russula pallidula*	RITF3331	Yunnan, southwestern China	MH027959	MH027961	Chen et al. (2019) and Chen et al. (2021a)
*Russula parvovirescens*	SDRM 6280	United States	MK532789	—	Unpublished
*Russula phloginea*	CNX530524068	Yunnan, southwestern China	MK860701	MK860704	Song et al. (2019)
*Russula phloginea*	CNX530524304	Yunnan, southwestern China	MK860700	MK860703	Song et al. (2019)
*Russula prasina*	HMAS 281232	Guangxi, southern China	MH454351	—	Hyde et al. (2019)
*Russula prasina*	HMAS 279806	Guangxi, southern China	MH454353	—	Unpublished
*Russula prasina*	HMAS 279805	Guangxi, southern China	MH454352	—	Unpublished
*Russula pseudobubalina*	GDGM70632	Guangdong, southern China	MF433036	—	Li et al. (2019)
*Russula* sp.	Pj3-mOTU063	Japan	LC260471	—	Murata and Nara (2017)
*Russula* sp.	Pa1-mOTU086	Japan	LC315895	—	Murata and Nara (2017)
*Russula* sp.	TY613	Japan	LC367995	—	Miyamoto et al. (2018)
*Russula* sp.	Pj3-mOTU065	Japan	LC260473	—	Murata and Nara (2017)
*Russula* sp.	HMAS:279584	China	MG719936	—	Li et al. (2018)
*Russula* sp.	HMAS 276811	China	LT602970	LT602947	Unpublished
*Russula* sp.	6 MAS-2010	Japan	GQ359820	—	Motomura et al. (2010)
*Russula* sp.	B4-1	Japan	LC553324	—	Yamato et al. (2021)
*Russula* sp.	dc264	Japan	LC538091	—	Ishikawa et al. (2020)
*Russula* sp.	TJS2020-03	China	OM281259	OM281030	Unpublished
*Russula* sp.	TYY-73	China	OK584446	—	Unpublished
*Russula* sp.	1734	Hunan, central China	AB769908	—	Huang et al. (2014)
*Russula* sp.	HMAS:271715	China	KX441239	KX441486	Unpublished
***Russula subatropurpurea***	**N.K. Zeng4898 (FHMU4841)**	**Hainan, southern China**	**OP837465**	**—**	**Present study**
***Russula subatropurpurea***	**N.K. Zeng4910 (FHMU4854)**	**Hainan, southern China**	**OP837467**	**OP837457**	**Present study**
***Russula subatropurpurea***	**N.K. Zeng5034 (FHMU4812)**	**Hainan, southern China**	**OP837468**	**OP837458**	**Present study**
***Russula subatropurpurea***	**N.K. Zeng4764 (FHMU5454)**	**Hainan, southern China**	**OP837466**	**—**	**Present study**
*Russula subatropurpurea*	K16080818	Guangdong, southern China	MF433038	—	Li et al. (2019)
*Russula subatropurpurea*	K16080816	Guangdong, southern China	MF433037	—	Li et al. (2019)
*Russula subatropurpurea*	K17071401	Guangdong, southern China	MH422579	—	Li et al. (2019)
*Russula subbubalina*	RITF4710	Guangdong, southern China	MW646978	MW646990	Chen et al. (2021a)
*Russula subbubalina*	RITF4715	Guangdong, southern China	MW646979	MW646991	Chen et al. (2021a)
*Russula subpallidirosea*	RITF4083	Guangdong, southern China	MK860697	MK860702	Song et al. (2019)
*Russula subpunicea*	RITF3715	Guangxi, southern China	MN833635	MW397124	Chen et al. (2021b)
*Russula subpunicea*	RITF2648	Zhejiang, eastern China	MN833638	MW397125	Chen et al. (2021b)
*Russula subpunicea*	RITF1435	Hunan, central China	MN833637	MW397126	Chen et al. (2021b)
*Russula subpunicea*	RITF2615	Hunan, central China	MN833636	MW397127	Chen et al. (2021b)
*Russula substriata*	XHW4766	Yunnan, southwestern China	MH724921	MH714540	Wang et al. (2019)
*Russula variata*	BPL241	United States	KT933959	KT933818	Looney et al. (2016)
*Russula vesca*	RITF5038	China	MW646984	—	Chen et al. (2021a)
*Russula vesca*	BPL284	United States	KT933978	KT933839	Looney et al. (2016)
*Russula vesca*	AT2002091	Sweden	DQ422018	—	Buyck et al. (2008)
*Russula violeipes*	BB 07.273	Slovakia	—	KU237534	Buyck et al. (2018)
*Russula violeipes*	SFC20121010-06	South Korea	KF361808	KF361858	Park et al. (2013)
*Russula virescens*	HJB9989	Belgium	DQ422014	—	Buyck et al. (2008)
*Russula viridicinnamomea*	K15091418	Guangdong, southern China	MK049972	—	Yuan et al. (2019)
*Russula viridicinnamomea*	RITF3324	China	MW397098	MW397130	Unpublished
*Russula viridirubrolimbata*	HBAU 15011	Hunan, central China	MT337526	—	Deng et al. (2020)
*Russula werneri*	IB1997/0786	Europe	DQ422021	—	Unpublished
*Russula xanthovirens*	GDGM 71145	Guangdong, southern China	MG786056	—	Song et al. (2018b)
***Russula xanthovirens***	**N.K. Zeng3025 (FHMU1986)**	**Hainan, southern China**	**—**	**OP837452**	**Present study**
***Russula xanthovirens***	**N.K. Zeng3041 (FHMU2002)**	**Hainan, southern China**	**MT822963**	**MT829148**	**Present study**
*Russula xanthovirens*	B17091630	Guangdong, southern China	MG786055	—	Unpublished

GenBank numbers in bold indicate the newly generated sequences.

### Dataset assembly

A total of 28 DNA sequences (10 28S, 10 ITS, and 8 *TEF1*) from 12 collections were newly generated. Edited sequences were deposited in GenBank; the GenBank accession numbers of 28S and ITS are listed in Table 1, and eight *TEF1s* are presented here [N.K. Zeng3025 (FHMU1986): OP830898; N.K. Zeng3041 (FHMU2002): OP830899; N.K. Zeng4898 (FHMU4841): OP830900; N.K. Zeng4910 (FHMU4854): OP830901; N.K. Zeng5034 (FHMU4812): OP830902; N.K. Zeng4764 (FHMU5454): OP830903; N.K. Zeng4895 (FHMU4847): OP830904; and N.K. Zeng4968 (FHMU5535): OP830905]. For the concatenated dataset, 28S and ITS sequences from new collections were aligned with sequences from related taxa of subg. *Heterophyllidiae* (Table 1). *Russula maguanensis* and *R. substriata* were chosen as out-group referred from Chen et al. (2021a,b). Sequences were aligned using MUSCLE (Edgar, 2004) separately to test for phylogenetic conflict. Then, the sequences of the two genes were concatenated using Phyutility v2.2 for further analyses (Smith and Dunn, 2008).

### Phylogenetic analyses

Maximum likelihood (ML) and Bayesian inference (BI) were employed for phylogenetic analysis. ML analysis was conducted with the program RAxML 7.2.6 (Stamatakis, 2006) running 1,000 replicates combined with an ML search. Bayesian analysis with MrBayes 3.1 (Huelsenbeck and Ronquist, 2005) implementing the Markov Chain Monte Carlo (MCMC) technique and parameters predetermined with MrModeltes 2.3 (Nylander, 2004) was performed. The best-fit likelihood models for 28S and ITS were GTR + I + G and GTR + I + G, respectively. Bayesian analysis was repeated for 3.5 million generations and sampled every 100. Trees sampled from the first 25% of the generations were discarded as burn-in, and Bayesian posterior probabilities (PP) were then calculated for a majority consensus tree of the retained Bayesian trees. At the end of the run, the average deviation of split frequencies was 0.008640.

## Results

### Molecular data

**Figure 1:**
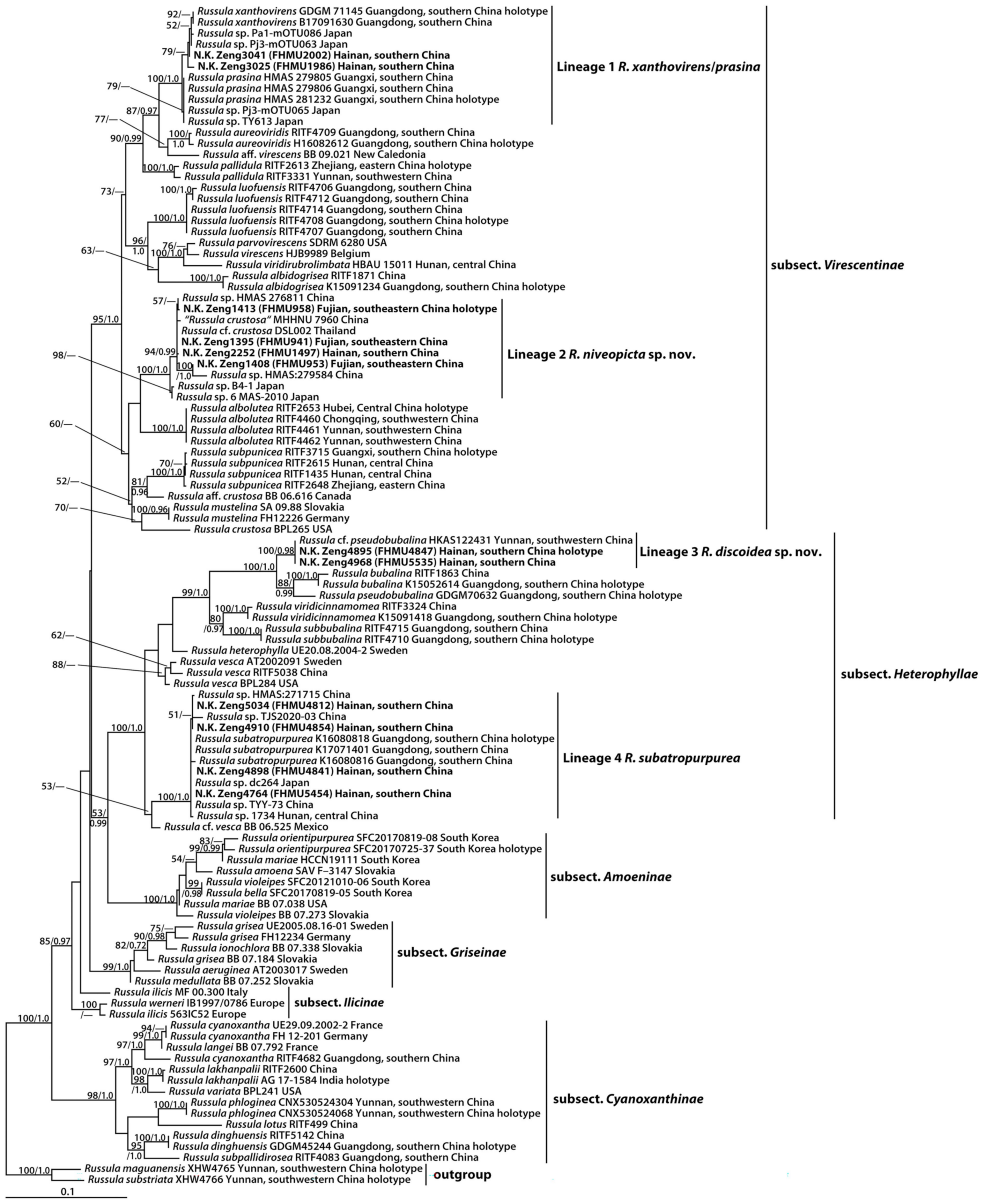
Phylogram of *Russula* subg. *Heterophyllidiae* inferred from a two-locus (rDNA 28S and ITS) dataset using RAxML. BS ≥ 50% and PP ≥ 0.95 are indicated above or below the branches as RAxML BS/PP.

The two-locus dataset (28S + ITS) consisted of 107 taxa and 1,601 nucleotide sites, and the alignment was submitted to TreeBASE (S30038). The topologies generated from ML and BI analyses were identical, though statistical support for some branches showed slight differences. The ML phylogram with branch lengths inferred from the 28S and ITS dataset is shown in Figure 1.

The phylogeny indicated that our new collections of subg. *Heterophyllidiae* were grouped into four independent lineages (1–4) (Figure 1). Lineage 1, with strong statistical support (BS = 100%, PP = 1.0), included the holotype (GDGM 71145) of *R. xanthovirens* Y. Song and L.H. Qiu, the holotype (HMAS 281232) of *R. prasina* G.J. Li and R.L. Zhao, one specimen (B17091630) identified as *R*. *xanthovirens*, two collections (HMAS 279805 and HMAS 279806) identified as *R. prasina*, four unidentified *Russula* collections (Pa1-mOTU086, Pj3-mOTU063, Pj3-mOTU065, and TY613), and two new collections (FHMU1986 and FHMU2002); lineage 2, with high statistical support (BS = 100%, PP = 1.0), was comprised of four new specimens (FHMU958, FHMU941, FHMU1497, and FHMU953), five unidentified *Russula* collections (HMAS276811, HMAS279584, B4-1, 6 MAS-2010, and DSL002), and one specimen (MHHNU 7960) labeled as *R. crustosa* Peck; lineage 3, with strong statistical support (BS = 100%, PP = 0.98), included two new collections (FHMU4847 and FHMU5535) and one specimen (HKAS122431) labeled as *R.* cf. *pseudobubalina* J.W. Li and L.H. Qiu; lineage 4, with strong statistical support (BS = 100%, PP = 1.0), was comprised of the holotype (K16080818) of *R. subatropurpurea* J.W. Li and L.H. Qiu, two specimens (K17071401 and K16080816) identified as *R. subatropurpurea*, five unidentified *Russula* specimens (HMAS:271715, TJS2020-03, dc264, TYY-73, and 1734), and four new specimens (FHMU4812, FHMU4841, FHMU4854, and FHMU5454) (Figure 1).

### Taxonomy

***Russula discoidea*** N.K. Zeng, Y.X. Han, and Zhi Q. Liang, sp. nov.

**Figure 2:**
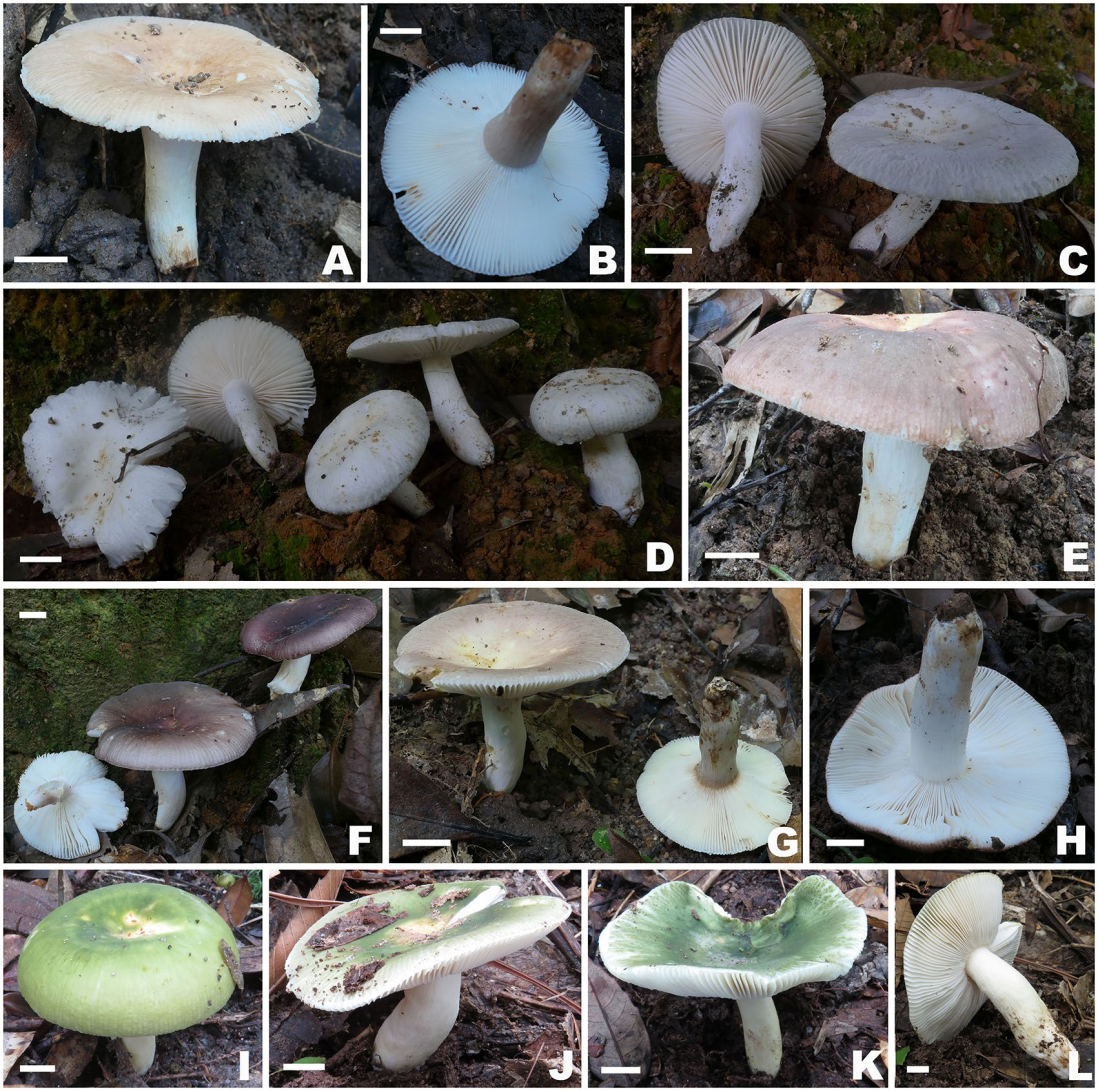
Basidiomata of *Russula* subg. *Heterophyllidiae* species. **(A,B)** *Russula discoidea* (FHMU4847, holotype); **(C,D)** *Russula niveopicta* (FHMU958, holotype); **(E–H)** *R. subatropurpurea* **(E,H)** FHMU5454; **(F)** FHMU4812; **(G)** FHMU4841; **(I–L)** *R. xanthovirens* **(I,K)** FHMU2002; **(J,L)** FHMU1986; scale bars = 1 cm; photographs: N. K. Zeng.

**Figure 3:**
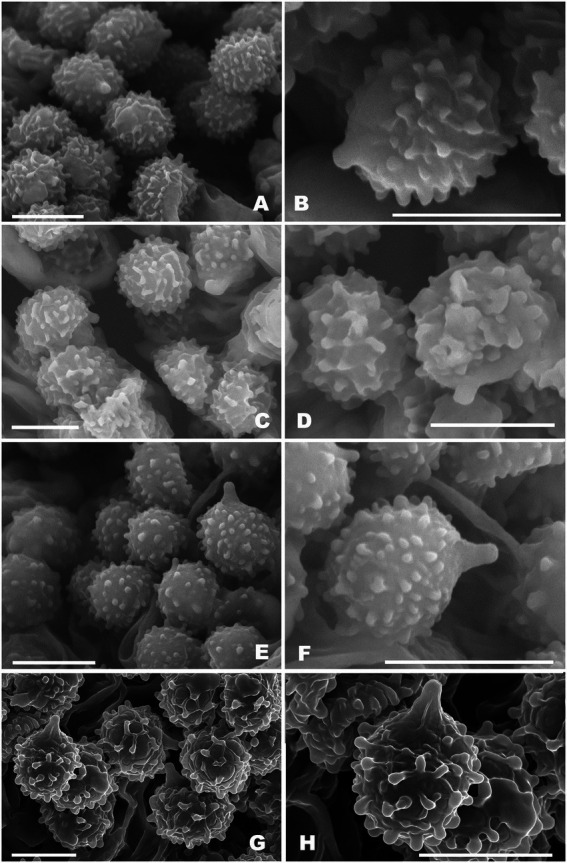
Basidiospores of *Russul*a subg. *Heterophyllidiae* species from herbarium materials under SEM. **(A,B)** *Russula discoidea* (FHMU4847, holotype); **(C,D)** *Russula niveopicta* (FHMU958, holotype); **(E,F)** *R. subatropurpurea* (FHMU5454); **(G,H)** *R. xanthovirens* (FHMU1986); scale bars = 5 μm; photographs: Y. X. Han.

**Figure 4:**
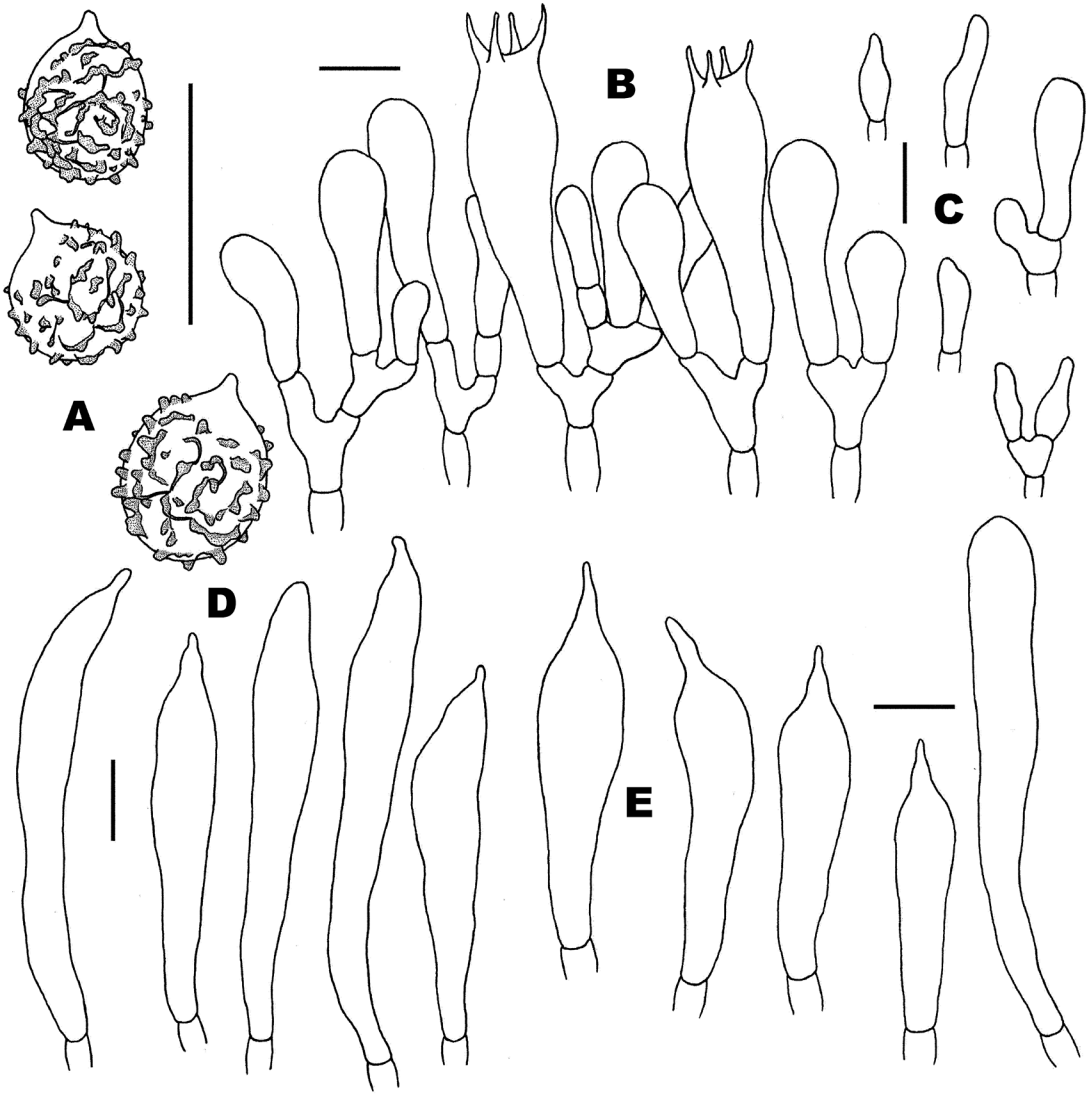
Microscopic features of *Russula discoidea* (FHMU4847, holotype). **(A)** Basidiospores. **(B)** Basidia and basidiola. **(C)** Marginal cells. **(D)** Pleurocystidia. **(E)** Cheilocystidia. Scale bars = 10 μm. Drawings by Y. X. Han.

**Figure 5:**
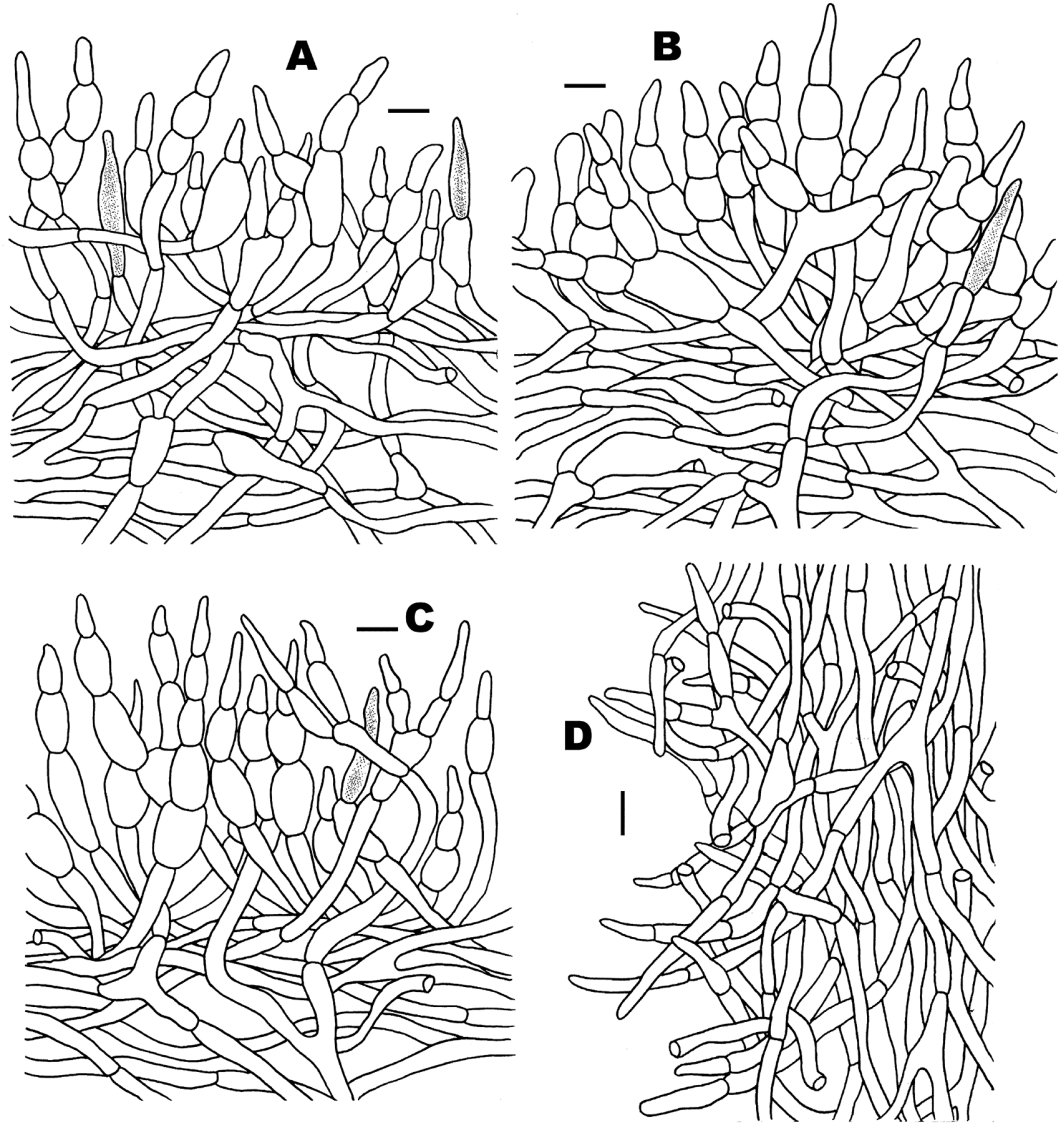
Microscopic features of *Russula discoidea* (FHMU4847, holotype). **(A)** Pileipellis at pileus center. **(B)** Pileipellis at the middle part between the center and margin of the pileus. **(C)** Pileipellis at pileus margin. **(D)** Stipitipellis. Scale bars = 10 μm. Drawings by Y. X. Han.

Figures 2A,B, 3A,B, 4, 5.

MycoBank: MB846471.

Diagnosis: Differs from closest species of *R*. subg. *Heterophyllidiae* by a cinnamon buff pileus, occasionally forked lamellae, basidiospores with small crests and ridges (0.3–0.7 μm high) forming an incomplete reticulum, cystidia slightly becoming yellowish brown in SV, and it is associated with fagaceous trees.

Etymology: Latin “*discoidea*” refers to the discoid pileus.

Holotype: CHINA. Hainan Province: Wanning County, Bofangling, elev. 80 m, 29 August 2020, *N.K. Zeng4895* (FHMU4847).

**Basidiomata** medium-sized. **Pileus** 5.6–6.8 cm in diameter, convex to applanate, center slightly depressed, margin occasionally cracked; surface dry, cinnamon buff (7A2), margin with radial tuberculate-striate; context 4.5–7 mm thick at the center of the pileus, white (3A1), unchanging in color when injured. **Hymenophore** lamellate adnate; lamellae 3.5–4 mm in height, occasionally forked, white (3A1), unchanging in color when injured; lamellulae common, concolorous with lamellae. **Stipe** 3.6–4.5 × 1.1 cm, central, subcylindric to cylindric; surface dry, white (3A1) to cinnamon buff (7B4). **Odor** indistinct. **Spore print** not obtained.

**Basidiospores** (excluding ornamentation) [40/2/2] 5–6.1–7(−7.5) × 4–5–6(−6.5) μm, Q = 1.0–1.5(−1.75), Qm = 1.21 ± 0.15, globose to ellipsoid, ornamentation composed of relatively small, dense (8–10 in a 3 μm diameter circle), amyloid, subcylindrical warts, 0.3–0.7 μm high, isolated or rarely fused (0–3 fusions in the circle), small crests and ridges forming an incomplete reticulum, connected by occasional line connections (1–3 in the circle); suprahilar spot inamyloid. **Basidia** 26.5–35–38.5 × 9–10.5–11 μm, hyaline in KOH, thin- to slightly thick-walled (0.4–0.5 μm), clavate to subcylindrical, four-spored; sterigmata 4–6 μm, slightly tortuous, sometimes straight; basidiola cylindric, then narrowly clavate, *ca.* 4–8.5 μm wide. **Pleurocystidia** numerous, *ca.* 1,800/mm^2^, 46.5–57–66.5 × 5.5–7–9(−10.5) μm, narrowly clavate to subcylindrical, apex often obtuse or acute, sometimes moniliform, occasionally with 2–6 μm long appendage, thin- to slightly thick-walled (0.4–0.5 μm); contents granulose, yellowish in Congo Red, slightly becoming yellowish brown in SV. **Cheilocystidia** 36–41–57(−63.5) × 7.5–9–10.5 μm, fusiform to subcylindrical, apex obtuse or mucronate, sometimes with 5–9 μm long appendage, slightly thick-walled (up to 0.5 μm); contents granulose, yellowish in Congo Red, slightly becoming yellowish brown in SV. **Lamellae edges** fertile. **Marginal cells** (11–)12–15.5–19 × (3.5–)4–5–6.5 μm, clavate or subcylindrical, usually shorter than basidioles, thin- to slightly thick-walled (up to 0.4 μm). **Lamellar trama** mainly composed of spherocytes measuring up to 38 μm in diameter, hyaline in KOH, slightly thick-walled (up to 1 μm). **Pileipellis** orthochromatic in Cresyl Blue, sharply delimited from the underlying context, 100–180 μm thick, two-layered, weakly gelatinized; composed of suprapellis (75–100 μm thick) and subpellis (30–80 μm thick). **Suprapellis** composed of erect to suberect hyphae 4–11 μm in diameter, thin-walled (up to 0.4 μm). **Subpellis** composed of horizontally oriented, 3–10 μm wide intricate hyphae. **Acid-resistant incrustations** absent. **Hyphal terminations near the pileus margin** sometimes branched, not flexuous, thin-walled (up to 0.4 μm); terminal cells 10–17.5–22 × 3.5–4–4.5 μm, narrowly subcylindrical or tapering upward; subterminal cells often subcylindrical to slightly inflated, occasionally branched. **Hyphal terminations on the middle part between the center and margin of pileus** sometimes branched and not flexuous; terminal cells 10–16.5–25(−40) × (3.5–)4.5–6–7 μm, attenuate subcylindrical; subterminal cells often subcylindrial to slightly inflated, occasionally branched. **Hyphal terminations near the pileus center** sometimes branched and not flexuous; terminal cells (8–)11.5–17–21 × 3.5–4–5.5(−6) μm, narrowly subcylindrical or tapering upward; subterminal cells often subcylindrial to slightly inflated, occasionally branched. **Pileal trama** composed of hyphae up to 30 μm in diameter, slightly thick-walled (up to 1 μm), hyaline in KOH. **Pileocystidia near the pileus margin** one-celled, 25–27.5–31 × 6–7–7.5 μm, cylindrical to clavate, apex usually obtuse, contents granulose, yellow in Congo Red slightly becoming yellowish brown in SV. **Pileocystidia near the pileus center** cylindrical to clavate, one-celled, 25–29–34.5 × 5–5.5–6 μm, contents granulose, yellow in Congo Red slightly becoming yellowish brown in SV. **Cystidioid hyphae** in subpellis and context, contents granulose. **Stipitipellis** a cutis, composed of hyphae thin- to slightly thick-walled (up to 0.4 μm), 3–7 μm wide, hyaline in KOH; terminal cells 9–38 × 3.5–5.5 μm, subcylindrical, or subclavate. **Stipe trama** mainly composed of spherocytes measuring up to 32 μm in diameter, hyaline in KOH, thick-walled (1–1.5 μm). **Clamp connections** are absent in all tissues.

Habitat: Solitary on the ground in forests dominated by fagaceous trees.

Known distribution: Southern China (Hainan Province).

Additional specimen examined: CHINA. Hainan Province: Changjiang County, Bawangling National Nature Reserve, elev. 650 m, 3 September 2020, *N.K. Zeng4968* (FHMU5535).

Notes: Phylogenetically, our new species *R. discoidea* is closely related to *R. bubalina* J.W. Li and L.H. Qiu and *R. pseudobubalina* J.W. Li and L.H. Qiu (Figure 1). However, *R. bubalina*, originally described in Guangdong Province of southern China, has a smaller basidioma (pileus 3.5–5.4 cm in diameter), basidiospores with ornamentations composed of subcylindrical warts and not forming reticulum (Li et al., 2019); *R. pseudobubalina*, also described from Guangdong Province of southern China, has a smaller basidioma (pileus 3.1–4.6 cm in diameter), an absence of forked lamellae, basidiospores with ornamentations composed of subcylindrical warts, not forming a reticulum, and uninflated subterminal cells in the pileipellis (Li et al., 2019). Moreover, sequence comparison of the newly generated ITS sequences *via* BLAST showed that the new species *R. discoidea* was most closely related to a collection labeled as *R.* cf. *pseudobubalina* (HKAS122431) (99.04% similarity) from China, a specimen also labeled as *R*. cf. *pseudobubalina* (DSL001) (96.41%) from Thailand, a collection labeled as *R*. sp. (YM25) (95.48%) from Japan, a material labeled as *R*. sp. (YM220) (95.20%) from Japan, and a collection labeled as *R*. sp. (YM4589) (95.20%) from Japan.

Morphologically, *R. discoidea* may be confused with *R. subbubalina* B. Chen and J.F. Liang, a recently described species from Guangdong Province of southern China. However, *R. subbubalina* has a larger basidioma (pileus 5–10 cm in diameter), a dark salmon pileus with rusty spots when young and pruina in some parts, the striation on pileus is inconspicuous, pleurocystidia, cheilocystidia, and pileocystidia near the pileus margin turning reddish black in SV, and pileocystidia near the pileus center turning reddish in SV (Chen et al., 2021a).

***Russula niveopicta*** N.K. Zeng, Y.X. Han and Zhi Q. Liang, sp. nov.

**Figure 6:**
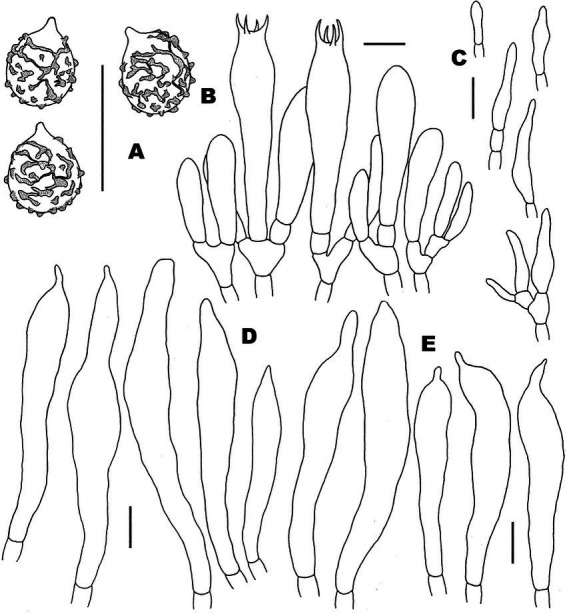
Microscopic features of *Russula niveopicta* (FHMU958, holotype). **(A)** Basidiospores. **(B)** Basidia and basidiola. **(C)** Marginal cells. **(D)** Pleurocystidia. **(E)** Cheilocystidia. Scale bars = 10 μm. Drawings by Y. X. Han.

**Figure 7:**
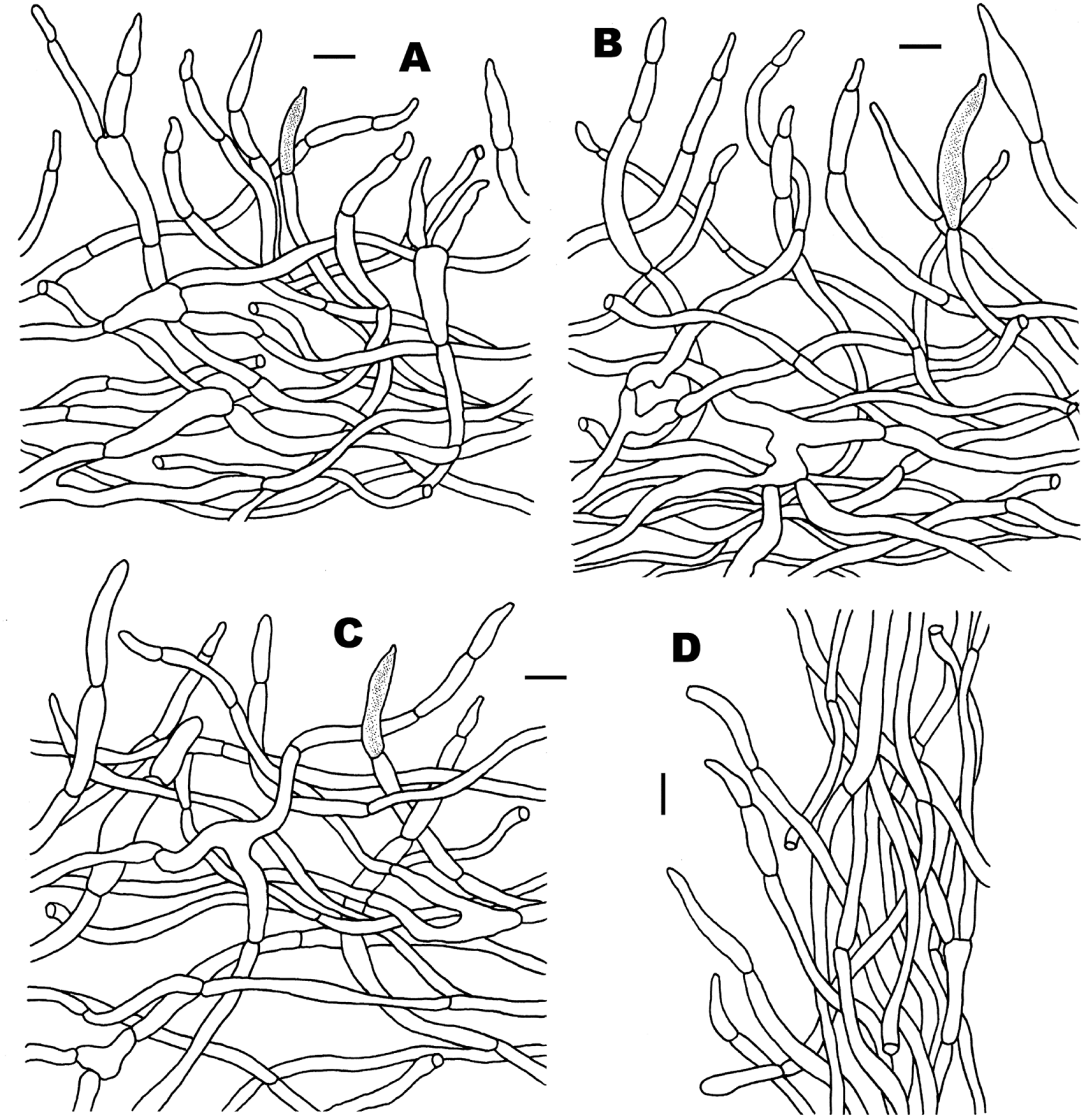
Microscopic features of *Russula niveopicta* (FHMU958, holotype). **(A)** Pileipellis at pileus center. **(B)** Pileipellis at the middle part between the center and margin of the pileus. **(C)** Pileipellis at pileus margin. **(D)** Stipitipellis. Scale bars = 10 μm. Drawings by Y. X. Han.

Figures 2C,D, 3C,D, 6, 7.

MycoBank: MB846472.

Diagnosis: Differs from closest species of *R*. subg. *Heterophyllidiae* by a white pileus with white tuberculate-striate margin, forked lamellae, a white stipe, basidiospores with small crests and ridges (0.4–0.7 μm) forming an incomplete reticulum, cystidia slightly becoming yellowish brown in SV, and it is associated with fagaceous trees.

Etymology: Latin “*niveopicta*” refers to the pileus with the white tuberculate-striate margin.

Holotype: CHINA. Fujian Province: Zhangping County, Xinqiao Town, Chengkou Village, elev. 350 m, 13 August 2013, *N.K. Zeng1413* (FHMU958).

**Basidiomata** small- to medium-sized. **Pileus** 3.5–5.5 cm diameter, convex to applanate, center slightly depressed, margin occasionally cracked; surface dry, white (2A1), margin with white radial tuberculate-striate; context 3–5 mm thick at the center of the pileus, white (3A1), unchanging in color when injured. **Hymenophore** lamellate, adnate; lamellae 2–5 mm in height, occasionally forked, white (3A1), unchanging in color when injured; occasionally with lamellulae, concolorous with lamellae. **Stipe** 3–4.5 × 0.8–1.3 cm, central, subcylindric to cylindric, hollow; surface white (3A1), with finely longitudinally white veins. **Odor** indistinct. **Spore print** not obtained.

**Basidiospores** (excluding ornamentation) [100/5/4] 5–6.2–7(−8) × 4.5–5.3–6(−6.5) μm, Q = 1–1.3(−1.4), Qm = 1.16 ± 0.10, globose to broadly ellipsoid, ornamentation composed of relatively small, moderately distant to dense (6–8 in a 3 μm diameter circle) amyloid, subcylindrical warts, 0.4–0.7 μm high, isolated or rarely fused (0–2 fusions in the circle), small crests and ridges forming an incomplete reticulum, connected by occasional line connections [(0–)1–3 in the circle]; suprahilar spot inamyloid. **Basidia** (38–)40–49.5–53 × 9–10.5–11.5(−12) μm, hyaline in KOH, slightly thick-walled (0.5 μm), clavate, four-spored; sterigmata 4–5 μm, slightly tortuous, sometimes straight; basidiola cylindric, then narrowly clavate, *ca.* 4.5–11 μm wide. **Pleurocystidia** numerous, *ca.* 2,600/mm^2^, (45.5–)66–73.5–81 × 7–10–11.5(−12.5) μm, clavate to subcylindrical, apex often mucronate, sometimes moniliform, occasionally with 2–5 μm long appendage, slightly thick-walled (up to 0.5 μm); contents granulose, yellowish in Congo Red, slightly becoming yellowish brown in SV. **Cheilocystidia** 46–55.5–65(−69.5) × 7.5–9–10.5 μm, clavate to subcylindrical, apex obtuse or mucronate, sometimes with 3–9 μm long appendage, slightly thick-walled (up to 0.5 μm); contents granulose, yellowish in Congo Red, slightly becoming yellowish brown in SV. **Lamellae edges** fertile. **Marginal cells** (10–)16.5–20–25 × 4–4.5–5 μm, clavate or subcylindrical, usually shorter than basidioles, thin-walled (up to 0.4 μm). **Lamellar trama** mainly composed of spherocytes measuring up to 38 μm in diameter, hyaline in KOH, slightly thick-walled (up to 1 μm). **Pileipellis** orthochromatic in Cresyl Blue, sharply delimited from the underlying context, 190–270 μm thick, two-layered, weakly gelatinized; composed of suprapellis (70–100 μm thick) and subpellis (125–180 μm thick). **Suprapellis** composed of erect to suberect hyphae 3–8 μm in diameter, slightly thick-walled (up to 0.5 μm). **Subpellis** composed of horizontally oriented, 3.5–9 μm wide intricate hyphae. **Acid-resistant incrustations** absent. **Hyphal terminations near the pileus margin** not flexuous, slightly thick-walled (up to 0.5 μm); terminal cells (12–)15–20.5–31 × 3.5–4–5 μm, narrowly subcylindrical; subterminal cells often wider, unbranched. **Hyphal terminations on the middle part between the center and margin of pileus** unbranched and not flexuous; terminal cells 16–22.5–27.5(−32) × (3–)3.5–4–5.5 μm, subcylindrical; subterminal cells often wider, unbranched. **Hyphal terminations near the pileus center** branched and not flexuous; terminal cells (8–)15–17.5–21(−22) × 4–5–5.5 μm, mainly clavate, occasionally subcylindrical; subterminal cells subcylindrical, sometimes branched. **Pileal trama** composed of hyphae up to 38 μm in diameter, slightly thick-walled (up to 1 μm), hyaline in KOH. **Pileocystidia near the pileus margin** one-celled, 28–35.5–42 × 4.5–5–5.5 μm, cylindrical to clavate, apex usually mucronate, contents granulose, yellow in Congo Red, slightly becoming yellowish brown in SV. **Pileocystidia near the pileus center** cylindrical to clavate, one-celled, 21–38.5–47 × 5–6–6.5(−7) μm, contents granulose, yellow in Congo Red, slightly becoming yellowish brown in SV. **Cystidioid hyphae** in subpellis and context, contents granulose. **Stipitipellis** a cutis, composed of hyphae thin-walled (up to 0.4 μm), 3–8 μm wide, hyaline in KOH; terminal cells 16–32 × 3.5–5.5 μm, subcylindrical or subclavate. **Stipe trama** mainly composed of spherocytes measuring up to 40.5 μm in diameter, hyaline in KOH, slightly thick-walled (up to 1 μm). **Clamp connections** are absent in all tissues.

Habitat: Gregarious or solitary on the ground in forests dominated by trees of *Castanopsis* (D. Don) Spach.

Known distribution: Southern and southeastern China (Hainan and Fujian Provinces).

Additional specimens examined: CHINA. Fujian Province: Zhangping County, Xinqiao Town, Chengkou Village, elev. 350 m, 9 August 2013, *N.K. Zeng1395* (FHMU941); same location, 13 August 2013, *N.K. Zeng1408* (FHMU953); Hainan Province: Yinggeling of Hainan Tropical Rainforest National Park, elev. 700 m, 30 July 2015, *N.K. Zeng2252* (FHMU1497).

Notes: In China, our new species *R. niveopicta* was misidentified as *R. crustosa* (Figure 1), originally described in North America. However, *R. crustosa* has a yellowish brown pileus with defined patches, basidiospores with warty ornamentations, not forming a reticulum (Peck, 1886).

Morphologically, *R. niveopicta* may be confused with four species: *R. albidogrisea* J.W. Li and L.H. Qiu, *R. alboareolata* Hongo, *R. albolutea* B. Chen and J.F. Liang, and *R. pallidula* Bin Chen and J. F. Liang. However, the Chinese species *R. albidogrisea*, originally described in Guangdong Province of southern China, has basidiospores with lower ornamentations composed of conical to hemispherical wart (up to 0.4 μm high), forming an almost complete reticulum, and pleurocystidia, cheilocystidia, and pileocystidia unchanged in SV (Das et al., 2017). *Russula alboareolata*, originally described from Japan, has equal lamellae, inflated subterminal cells, and basidiospores with ornamentations tend to be almost a complete reticulum (Hongo, 1979); moreover, the molecular phylogeny based on the 28S dataset indicated that *R. niveopicta* is genetically distant from two collections of *R. alboareolata* from Japan (data not shown). *Russula albolutea*, originally described from the Hubei Province of central China, possesses a larger basidioma (pileus 5–7.5 cm in diameter), pleurocystidia, and cheilocystidia turning mauve in SV, and pileocystidia turning reddish in SV (Chen et al., 2021b). *Russula pallidula*, originally described from Zhejiang Province of eastern China, is distinct in its basidiospores with lower ornamentations composed of bluntly conical wart (up to 0.35 μm high), forming a partial reticulum, pleurocystidia dark gray in SV, and inflated subterminal cells in pileipellis (Chen et al., 2019).

Sequence comparison of the newly generated ITS sequences *via* BLAST showed that the new species *R. niveopicta* was most closely related to a collection labeled as *R.* sp. (HMAS:279584) (99.79%) from China, a specimen labeled as *R.* sp. (HMAS 276811) (99.68%) from China, a material misidentified as *R. crustosa* (MHHNU 7960) (99.38%) from China, a collection labeled as *R.* cf. *crustosa* (DSL002) (99.38%) from Thailand, and a specimen labeled as *R.* sp. (MAS-2010) (98.61%) from Japan.

***Russula subatropurpurea*** J.W. Li and L.H. Qiu, Phytotaxa 392 (4): 272, 2019.

**Figure 8:**
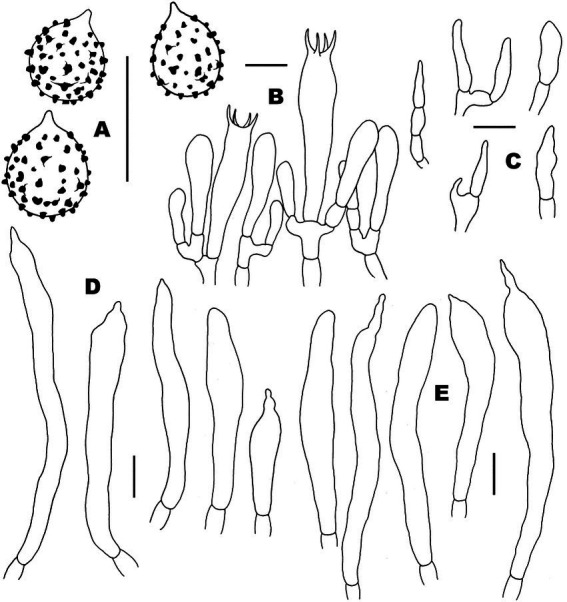
Microscopic features of *Russula subatropurpurea* (FHMU5454). **(A)** Basidiospores. **(B)** Basidia and basidiola. **(C)** Marginal cells. **(D)** Pleurocystidia. **(E)** Cheilocystidia. Scale bars = 10 μm. Drawings by Y. X.Han.

**Figure 9:**
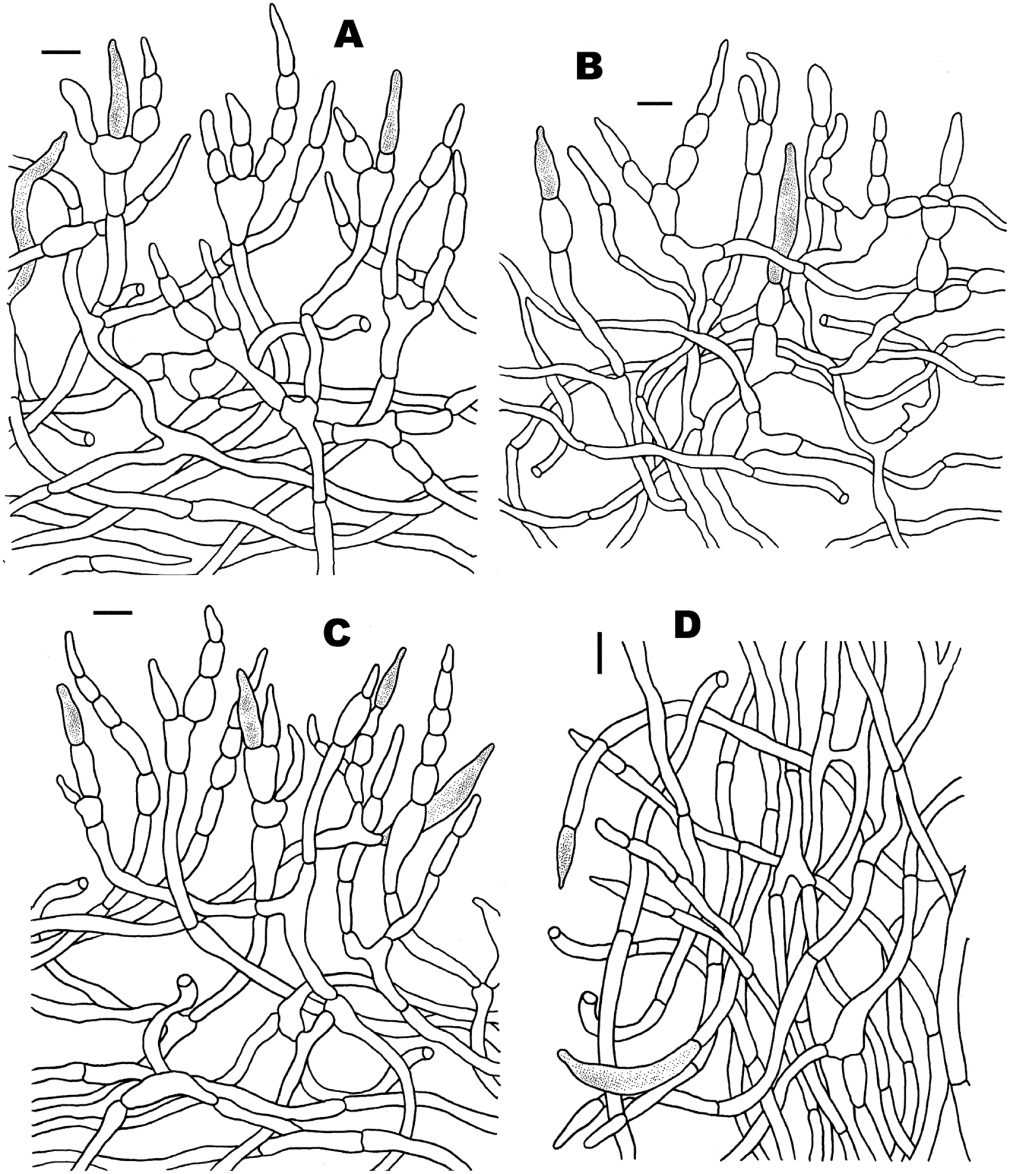
Microscopic features of *Russula subatropurpurea* (FHMU5454). **(A)** Pileipellis at pileus center. **(B)** Pileipellis at the middle part between the center and margin of the pileus. **(C)** Pileipellis at pileus margin. **(D)** Stipitipellis. Scale bars = 10 μm. Drawings by Y. X. Han.

Figures 2E–H, 3E,F, 8, 9.

**Basidiomata** small- to medium-sized. **Pileus** 4–6.5 cm in diameter, hemispherical at first, then applanate, center slightly depressed, margin occasionally cracked; surface dry, purplish brown (8F2), yellowish brown (2B3) to pale yellow (1A3) on pileus center, margin with radial tuberculate-striate; context 4–8 mm thick at the center of the pileus, white (2A1), unchanging in color when injured. **Hymenophore** lamellate, adnate; lamellae 2.5–5 mm in height, crowded, often forked, white (2A1), unchanging in color when injured; lamellulae absence. **Stipe** 2.8–5.3 × 0.9–1.5 cm, central, subcylindrical to cylindrical, slightly narrow toward base; surface white (4A1). **Odor** indistinct. **Spore print** not obtained.

**Basidiospores** (excluding ornamentation) [80/7/4] 5–6.1–7(−8) × 4–5.2–6(−6.5) μm, Q = 1–1.4(−1.5), Qm = 1.18 ± 0.11, globose to ellipsoid, ornamentation composed of relatively small, dense [(8–)9–13 in a 3 μm diameter circle], amyloid, subcylindrical warts, 0.3–0.5 μm high, isolated or occasionally fused (0–2 fusions in the circle), without line connections, never forming a reticulum; suprahilar spot inamyloid. **Basidia** (20–)24.5–28.5–32(−40) × (5–)5.5–7–8.5(−9) μm, hyaline in KOH, thin-walled (up to 0.4 μm), clavate to subcylindrical, four-spored; sterigmata 3–9 μm, slightly tortuous, sometimes straight; basidiola clavate, *ca.* 4–7 μm wide. **Pleurocystidia** numerous 2,400/mm^2^, (30–)48–60–80 × 5.5–7–9 μm, clavate to slender fusiform, most with mucronate to moniliformous, occasionally with 2.5–5 μm long appendage, slightly thick-walled (up to 0.5 μm); contents granulose, yellowish in Congo Red, slightly becoming yellowish brown in SV. **Cheilocystidia** 50–69–76 × (5.5–)6–7.5–8 μm, narrowly clavate to slender subcylindrical, apex obtuse or mucronate, sometimes with 3–9 μm long appendage, slightly thick-walled (up to 0.5 μm); contents granulose, yellowish in Congo Red, slightly becoming yellowish brown in SV. **Lamellae edges** fertile. **Marginal cells** (11–)11.5–15–17 × 3–4.5–5(−6) μm, clavate or subcylindrical, usually shorter than basidiola, and thin-walled (up to 0.4 μm). **Lamellar trama** mainly composed of spherocytes measuring up to 31 μm in diameter, hyaline in KOH, slightly thick-walled (up to 1 μm). **Pileipellis** orthochromatic in Cresyl Blue, sharply delimited from the underlying context, 270–350 μm thick, two-layered, weakly gelatinized; composed of suprapellis (125–170 μm thick) and subpellis (150–200 μm thick). **Suprapellis** composed of erect to suberect hyphae 2.5–9 μm in diameter, slightly thick-walled (up to 0.4 μm). **Subpellis** composed of horizontally oriented, 3–8 μm wide intricate hyphae. **Acid-resistant incrustations** absent. **Hyphal terminations near the pileus margin** sometimes branched, not flexuous, slightly thick-walled (up to 0.4 μm); terminal cells (7–)8–15–20 × 2.5–3–5 μm, mainly attenuate acicular to subcylindrial; subterminal cells often wider and slightly inflated, and branched. **Hyphal terminations on the middle part between the center and margin of pileus** less flexuous, sometimes branched, terminal cells (8–)12.5–18–22 × 3.5–4–5.5 μm, mainly clavate, occasionally attenuate, subcylindrical to acicular; subterminal cells often wider and slightly inflated, occasionally branched. **Hyphal terminations near the pileus center** not flexuous; terminal cells 7–12.5–20 × 4–4.5–5(−5.5) μm, attenuate subcylindrical to acicular; subterminal cells often wider and slightly inflated, sometimes branched. **Pileal trama** is made up of hyphae up to 41 μm in diameter, slightly thick-walled (up to 1 μm), hyaline to pale yellowish in KOH. **Pileocystidia near the pileus margin** always one-celled, (16–)17.5–26–37 × 4.5–6–9.5 μm, cylindrical to fusiform, apex occasionally obtuse or usually mucronate, contents yellow in Congo Red, slightly becoming yellowish brown in SV. **Pileocystidia near the pileus center** narrower cylindrical to clavate, one-celled, 22–34–45 × 4.5–5.5–6 μm, contents granulose, yellow in Congo Red, slightly becoming yellowish brown in SV. **Cystidioid hyphae** in subpellis and context, contents granulose. **Stipitipellis** a cutis composed of interwoven hyphae thin-walled (up to 0.4 μm), 3–7 μm wide, hyaline in KOH; terminal cells 10–22 × 3–4.5 μm, subcylindrical or subclavate. **Stipe trama** mainly composed of spherocytes measuring up to 32 μm in diameter, hyaline to pale yellowish in KOH, slightly thick-walled (up to 1 μm). **Clamp connections** are absent in all tissues.

Habitat: Gregarious or solitary on the ground in forests dominated by fagaceous trees.

Known distribution: Southern China (Guangdong and Hainan Provinces).

Specimens examined: CHINA. Hainan Province: Yinggeling of Hainan Tropical Rainforest National Park, elev. 650 m, 14 August 2020, *N.K. Zeng4764* (FHMU5454); same location, 4 September 2020, *N.K. Zeng5034* (FHMU4812); Wanning County, Bofangling, elev. 80 m, 29 August 2020, *N.K. Zeng4898* (FHMU4841); same location and date, *N.K. Zeng4910* (FHMU4854).

Notes: *Russula subatropurpurea* was originally described in the Guangdong Province of southern China (Li et al., 2019). In the present study, it was also found to distribute in Hainan Province, tropical China. The species was redescribed according to our new specimens, which is characterized by a purplish brown, yellowish brown to pale yellow pileus, forking lamellae, an absence of lamellulae, basidiospores usually with subcylindrical isolated warts (0.3–0.5 μm), never forming a reticulum, long pleurocystidia and cheilocystidia slightly becoming yellowish brown in SV, and it is associated with fagaceous trees. Moreover, we noted that the pileus color and the striate on the pileus margin were described as “whole pileus purplish brown,” and “absent,” respectively (Li et al., 2019), whereas the pileus of our collections is pale yellow on the center, and the striate on the pileal margin is present.

***Russula xanthovirens*** Y. Song and L.H. Qiu, Cryptogamie, Mycologie 39 (1): 135, 2018.

**Figure 10:**
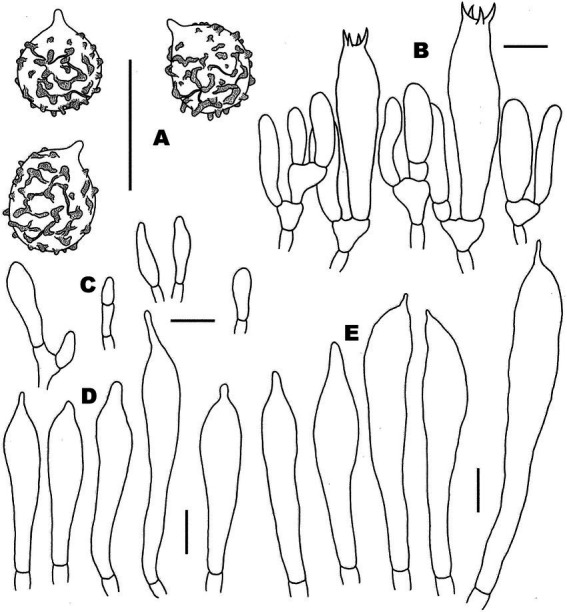
Microscopic features of *Russula xanthovirens* (FHMU1986). **(A)** Basidiospores. **(B)** Basidia and basidiola. **(C)** Marginal cells. **(D)** Pleurocystidia. **(E)** Cheilocystidia. Scale bars = 10 μm. Drawings by Y. X. Han.

**Figure 11:**
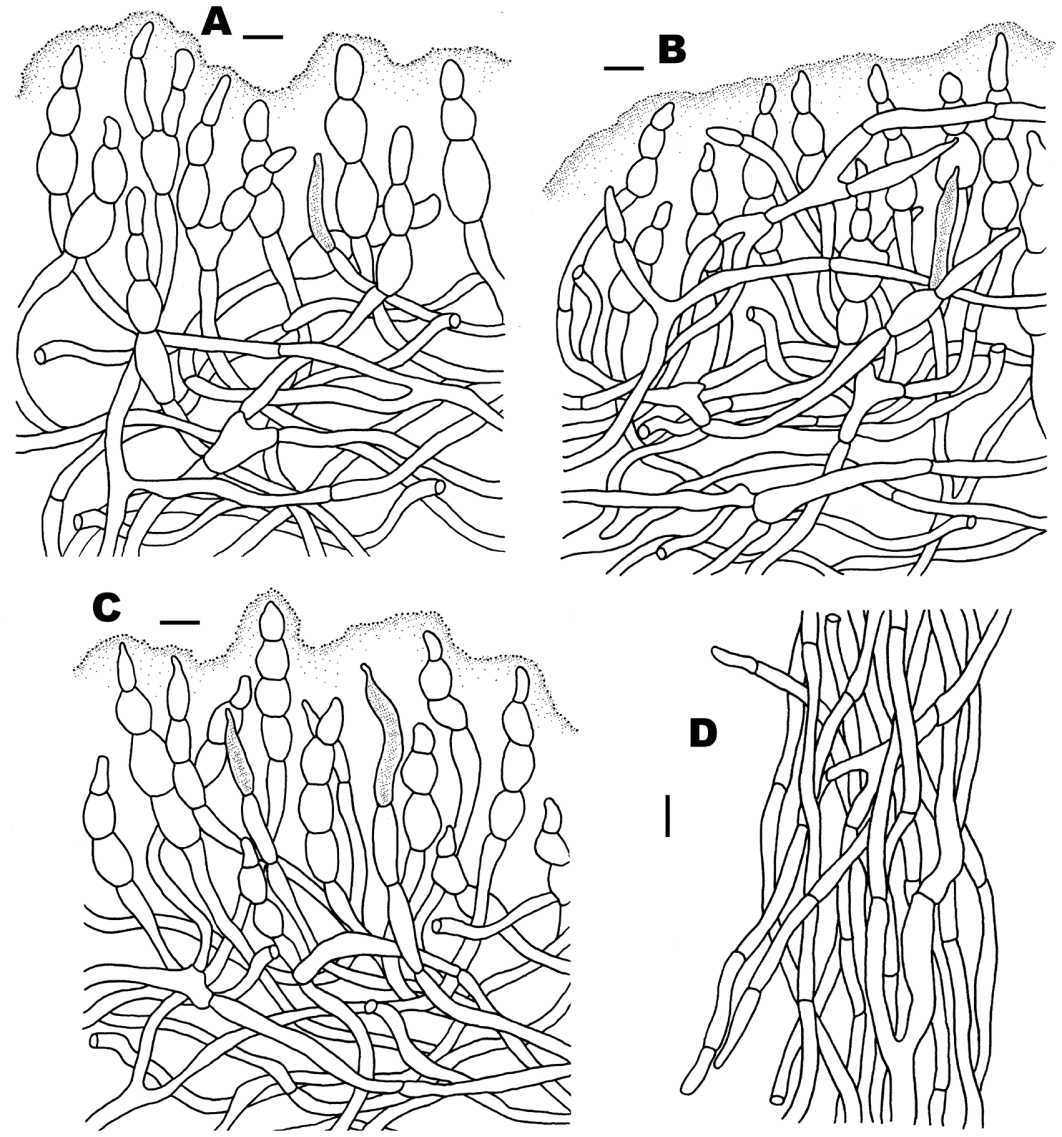
Microscopic features of *Russula xanthovirens* (FHMU1986). **(A)** Pileipellis at pileus center. **(B)** Pileipellis at the middle part between the center and margin of the pileus. **(C)** Pileipellis at pileus margin. **(D)** Stipitipellis. Scale bars = 10 μm. Drawings by Y. X. Han.

Figures 2I–L, 3G,H, 10, 11.

Synonym: *Russula prasina* G.J. Li and R.L. Zhao, Fungal Diversity 96: 215, 2019.

**Basidiomata** medium-sized. **Pileus** 6–7 cm in diameter, hemispherical at first, then applanate, center slightly depressed, cracked with age; surface dry, smooth, pale greenish (27A4) to dark greenish (27C6), with a pale yellowish center (3A3), margin with radial tuberculate-striate; context about 5 mm thick at the center of the pileus, white (2A1), unchanging in color when injured. **Hymenophore** lamellate, adnate; lamellae about 5 mm in height, crowded, often forked, white (2A1), unchanging in color when injured, lamellulae rare. **Stipe** 4.5–6.5 × 1–1.7 cm, central, subcylindrical to cylindrical; surface white (4A1), with striae. **Odor** indistinct. **Spore print** not obtained.

**Basidiospores** (excluding ornamentation) [40/2/2] 6–6.5–7 × 5–5.8–6.5 μm, Q = 1–1.3(−1.4), Qm = 1.11 ± 0.11, globose to broadly ellipsoid, ornamentation composed of relatively small, moderately distant to dense [(6–)7–8 in a 3 μm diameter circle] amyloid subcylindrical warts, 0.3–0.8 μm high, isolated or occasionally fused (0–2 fusions in the circle); small crests and ridges forming an incomplete reticulum, connected by occasional line connections [(0–)1–3 in the circle]; suprahilar spot inamyloid. **Basidia** (35–)39–42.5–45 × 10–10.5–11 μm, clavate to subcylindrical, hyaline in KOH, slightly thick-walled (up to 0.6 μm), clavate, four-spored; sterigmata 3–5 μm, slightly tortuous, sometimes straight; basidiola clavate, *ca.* 4.5–8 μm wide. **Pleurocystidia** moderately numerous, 1,100/mm^2^, (38–)41–52.5–62.5 × 8–8.5–9 μm, subcylindrical to fusoid, apically often obtuse or acute, occasionally with 3–8 μm long appendage, slightly thick-walled (up to 0.4 μm); contents granulose, yellowish in Congo Red, negative in SV. **Cheilocystidia** (47.5–)50–59–63.5(−88.5) × (8.5–)9.5–10–11.5 μm, clavate to fusoid, apex obtuse or mucronate, sometimes with 3–6 μm long appendage, slightly thick-walled (up to 0.4 μm); contents granulose, yellowish in Congo Red, negative in SV. **Lamellae edges** fertile. **Marginal cells** (6–)12–15–20 × 4–4.5–6(−6.5) μm, clavate or subcylindrical, usually shorter than basidiola, slightly thick-walled (up to 0.4 μm). **Lamellar trama** mainly composed of spherocytes measuring up to 38 μm in diameter, hyaline in KOH, slightly thick-walled (up to 1 μm). **Pileipellis** orthochromatic in Cresyl Blue, sharply delimited from the underlying context, 190–300 μm thick, two-layered, gelatinized; composed of suprapellis (110–170 μm thick) and subpellis (90–130 μm thick). **Suprapellis** composed of erect to suberect hyphae 3–10 μm in diameter, thin-walled (up to 0.4 μm). **Subpellis** composed of horizontally oriented, 2.5–9 μm wide intricate hyphae. Acid-resistant incrustations absent. **Hyphal terminations near the pileus margin** unbranched, not flexuous, thin-walled (up to 0.4 μm); terminal cells (9–)12–15.5–17 × 3.5–5–7 μm, subcylindrical to subulate; subterminal cells often wider, ellipsoid to globose. **Hyphal terminations on the middle part between the center and margin of pileus** not flexuous and unbranched, terminal cells (8–)18–21.5–28 × (3–)4–5–5.5 μm, subcylindrical to subulate; subterminal cells often wider, ellipsoid to globose. **Hyphal terminations near the pileus center** not flexuous; terminal cells 8–10.5–15 × 5–5.5–6.5(−7) μm, subcylindrical, apically obtuse; subterminal cells often wider, ellipsoid to subcylindrical, rarely branched. **Pileal trama** made up of hyphae up to 34.5 μm in diameter, thick-walled (up to 1 μm), hyaline to pale yellowish in KOH. **Pileocystidia near the pileus margin** one-celled, (22–)36–54.5–63 × 4–5–5.5 μm, cylindrical to clavate, apex occasionally obtuse or usually mucronate, contents yellow in Congo Red, unchanging in SV. **Pileocystidia near the pileus center** cylindrical to clavate, one-celled, (25–)30.5–36–40 × 4–4.5–5 μm, contents granulose, yellow in Congo Red, unchanging in SV. **Cystidioid hyphae** in subpellis and context, contents granulose. **Stipitipellis** a cutis composed of hyphae slightly thick-walled (up to 0.4 μm), 3–9 μm wide, hyaline in KOH; terminal cells 13–21 × 3.5–5 μm, subcylindrical or subclavate. **Stipe trama** mainly composed of spherocytes measuring up to 54 μm in diameter, hyaline to pale yellowish in KOH, slightly thick-walled (up to 1 μm). **Clamp connections** are absent in all tissues.

Habitat: Solitary on the ground in forests dominated by fagaceous trees.

Known distribution: Southern China (Guangdong and Hainan Provinces).

Specimens examined: CHINA. Hainan Province: Yinggeling of Hainan Tropical Rainforest National Park, elev. 650 m, 28 May 2017, *N.K. Zeng3025* (FHMU1986); same location, 29 May 2017, *N.K. Zeng3041* (FHMU2002).

Notes: *Russula xanthovirens* was originally described in the Guangdong Province of southern China (Song et al., 2018b); then, it was also reported from the Hainan Province, tropical China (Zeng and Jiang, 2020). The species was redescribed according to our new specimens, which is characterized by a greenish pileus, forking lamellae with rare lamellulae, basidiospores usually with small crests and ridges (0.3–0.8 μm), forming an incomplete reticulum, cystidia negative in SV, a two layers pileipellis, suprapellis with inflated subterminal cells, and it is associated with fagaceous trees.

The phylogenetic analyses showed that the holotype of *R. xanthovirens* and the holotype of *R. prasina* were in the same species-level lineage (Figure 1); moreover, there are no essential morphological differences between the two taxa (Song et al., 2018b; Hyde et al., 2019). We, therefore, treat *R. prasina* as a synonym of *R. xanthovirens*.

## Discussion

High species diversity of subg. *Heterophyllidiae* in China was revealed in previous/present studies, and 38 taxa of the subgenus have been described/reported in the country (Table 2). These taxa are members of sect. *Ingratae* (Quél.) Maire, subsect. *Cyanoxanthinae* Singer, subsect. *Griseinae* Jul. Schäff., subsect. *Heterophyllae* (Fr.) Jul. Schäff., subsect. *Substriatinae* X.H. Wang and Buyck, and subsect. *Virescentinae* Singer, respectively (Table 2). The combination of morphological features and phylogenetic analyses indicated that our new species *R. niveopicta* is a member of the subsect. *Virescentinae*, whereas *R. discoidea* belongs to the subsect. *Heterophyllae* (Figure 1). It is worth noting that *R. vesca* Fr., originally described in Europe, was reported to be distributed in China (Song, 2022); however, the Chinese collections identified as *R. vesca* are somewhat distant from European *R. vesca* in phylogenies (Figure 1; Song, 2022). The occurrence of *R. vesca* in China should be further defined in the future.

**Table 2:** Sections, subsections, and accepted species of *Russula* subgen. *Heterophyllidiae* in China.

Section	Subsection	Species	Locality	References
—	*Cyanoxanthinae* Singer	*R. dinghuensis* J.B. Zhang and L.H. Qiu	Guangdong, southern China	Zhang et al. (2017)
		*R. fusiformata* Yu Song	Guangdong, southern China	Song (2022)
		*R. lotus* Fang Li	Guangdong, southern China	Li and Deng (2018)
		*R. nigrovirens*	Yunnan, southwestern China	Zhao et al. (2015)
		*R. purpureorosea* Yu Song	Guangdong, southern China	Song (2022)
		*R. subpallidirosea* J.B. Zhang and L.H. Qiu	Guangdong, southern China	Zhang et al. (2017)
*Heterophyllae* Fr.	*Griseinae* Jul. Schäff.	*R. atroaeruginea*	Sichuan, southwestern China	Li et al. (2013)
	*Heterophyllae* (Fr.) Jul. Schäff.	*R. bubalina*	Guangdong, southern China	Li et al. (2019)
		*R. discoidea*	Hainan, southern China	Present study
		*R. pseudobubalina*	Guangdong, southern China	Li et al. (2019)
		*R. subatropurpurea*	Guangdong, southern China	Li et al. (2019)
		*R. subbubalina*	Guangdong, southern China	Chen et al. (2021a)
		*R. viridicinnamomea* F. Yuan and Y. Song	Guangdong, southern China	Yuan et al. (2019)
*Ingratae* (Quél.) Maire	—	*R. clavulus* B. Chen and J.F. Liang	Yunnan, southwestern China	Chen et al. (2021d)
		*R. gelatinosa* Y. Song and L.H. Qiu	Guangdong, southern China	Song et al. (2018a)
		*R. guangdongensis* Z.S. Bi and T.H. Li	Guangdong, southern China	Bi and Li (1986)
		*R. hainanensis*	Hainan, southern China	Han et al. (2022)
		*R. indocatillus* A. Ghosh, K. Das and R.P. Bhatt	India	Li et al. (2021)
		*R. multilamellula* B. Chen and J.F. Liang	Guizhou, southwestern China	Chen et al. (2021d)
		*R. pseudocatillus* F. Yuan and Y. Song	Guangdong, southern China	Yuan et al. (2019)
		*R. pseudopectinatoides*	Xizang, western China	Li et al. (2015)
		*R. punctipes* Singer	Hunan, central China	Song et al. (2018a)
		*R. rufobasalis* Y. Song and L.H. Qiu	Guangdong, southern China	Song et al. (2018a)
		*R. senecis*	Japan	Chen et al. (2014)
		*R. straminella* G.J. Li and C.Y. Deng	Guizhou, southwestern China	Li et al. (2021)
		*R. subpectinatoides* G.J. Li and Q.B. Sun	Jiangsu, eastern China	Li et al. (2021)
		*R. subpunctipes* J. Song	Hubei, central China	Song et al. (2020)
		*R. succinea* G.J. Li and C.Y. Deng	Guizhou, southwestern China	Li et al. (2021)
—	*Substriatinae* X.H. Wang and Buyck	*R. maguanensis*	Yunnan, southwestern China	Wang et al. (2019)
		*R. substriata*	Yunnan, southwestern China	Wang et al. (2019)
*Virescentinae* (Singer) Sarnari	*Virescentinae* Singer	*R. albidogrisea*	Guangdong, southern China	Das et al. (2017)
		*R. albolutea*	Hubei, central China	Chen et al. (2021b)
		*R. aureoviridis* Jing W. Li and L.H. Qiu	Guangdong, southern China	Das et al. (2017)
		*R. luofuensis* B. Chen and J.F. Liang	Guangdong, southern China	Chen et al. (2021a)
		*R. niveopicta*	Fujian, southeastern China	Present study
		*R. pallidula*	Zhejiang, eastern China	Chen et al. (2019)
		*R. subpunicea* B. Chen and J.F. Liang	Guangxi, southern China	Chen et al. (2021b)
		*R. viridirubrolimbata*	Guangxi, southern China	Ying (1983)
		*R. xanthovirens*	Guangdong, southern China	Song et al. (2018b)
—	—	*R. verrucospora* Y. Song and L.H. Qiu	Guangdong, southern China	Song et al. (2018b)

In China, most species of subg. *Heterophyllidiae* distribute in subtropical and tropical areas, only few taxa, namely *R. atroaeruginea* G.J. Li, Q. Zhao and H.A. Wen, *R. nigrovirens* Q. Zhao, Yang K. Li, and J. F. Liang, and *R. pseudopectinatoides* G. J. Li and H. A. Wen, occur in temperate areas (Li et al., 2013, 2015; Zhao et al., 2015). The geographical distribution pattern indicates that the subtropical–tropical region is the current species diversity center of subg. *Heterophyllidiae* in China.

Morphological characteristics used to define species of subg. *Heterophyllidiae* have been extensively discussed in previous studies (Chou and Wang, 2005; Li et al., 2013, 2015, 2018, 2019, 2021; Chen et al., 2014, 2019, 2021a,b,c,d; Zhao et al., 2015; Zhang et al., 2017; Li and Deng, 2018; Song et al., 2018a,b, 2020; Wang et al., 2019; Yuan et al., 2019; Han et al., 2022; Song, 2022). Ecological preference, also a useful feature to delimitate species, receives little attention. In the present study, our two new species *R. discoidea* and *R. niveopicta* are both associated with trees of Fagaceae Dumort. In addition to Fagaceae, we also noted that species of subg. *Heterophyllidiae* are associated with many other trees including Betulaceae Gray, Dipterocarpaceae Blume, Ericaceae Juss., Orchidaceae Juss., Pinaceae Spreng. ex F. Rudolphi, Rosaceae Juss., and Sterculiaceae (Candolle) Bartling (Das et al., 2013; Dutta et al., 2015; Zhao et al., 2015; Crous et al., 2017; Chen et al., 2021b). In China, together with our two new species, the vast majority of species of the subgenus such as *R. albolutea*, *R. clavulus*, *R. fusiformata*, *R. lotus*, *R. luofuensis*, *R. subbubalina*, *R. subpunctipes*, and *R. viridirubrolimbata* are associated with trees of Fagaceae (Ying, 1983; Li and Deng, 2018; Song et al., 2020; Chen et al., 2021a,b,d; Song, 2022); a great number of species including *R. atroaeruginea*, *R. indocatillus*, *R. multilamellula*, *R. pseudopectinatoides*, *R. straminella*, *R. subpectinatoides*, and *R. succinea* are associated with trees of Pinaceae (Li et al., 2013, 2015, 2021; Chen et al., 2021d); *R. hainanensis* is associated with trees of Dipterocarpaceae (Han et al., 2022); some species, e.g., *R. indocatillus* A. Ghosh, K. Das, and R. P. Bhatt, can be associated with both trees of Fagaceae and Pinaceae (Ghosh et al., 2020; Li et al., 2021). In addition, we also noted that *R. subpunicea* was reported to grow under trees of Betulaceae and Fagaceae (Chen et al., 2021b), and *R. nigrovirens* was found under trees of Ericaceae, Pinaceae, and Rosaceae (Zhao et al., 2015).

Recent phylogenetic studies have provided new insights into the phylogeny and geography of subg. *Heterophyllidiae* (Song et al., 2018b; Li et al., 2019; Chen et al., 2021a,b). Our phylogeny based on two-locus DNA sequences (28S + ITS) with 12 new specimens from southern China has contributed to new knowledge of subg. *Heterophyllidiae*. The phylogenetic analyses indicated that there are several clades having taxa from both sides of the Pacific, and allied species from China and North America are obvious (Figure 1). For example, Chinese *R. subpunicea* is closely related to one collection labeled as *R*. aff. *crustosa* from North America; one specimen identified as *R. parvovirescens* Buyck, D. Mitch., and Parrent from North America is affiliated with one material of *R.viridirubrolimbata* J.Z. Ying from China (Figure 1). The present study did not identify disjunct populations of the same purported taxon in the two regions (Figure 1). Similar scenarios have been documented for many other macrofungi (Halling, 2001; Zeng et al., 2013, 2016, 2017; Zhang et al., 2022a).

Biogeographic connections between China and Europe have been discussed in other macrofungi such as *Phylloporus* Quél., *Cantharellus* Adans. ex Fr., and *Craterellus* Pers. (Zeng et al., 2013; Wu et al., 2022; Zhang et al., 2022a,b). The geography of subg. *Heterophyllidiae* between the two regions was also noted, for example, one specimen identified as *R. virescens* (Schaeff.) Fr. from Europe is closely related to Chinese *R. viridirubrolimbata* (Figure 1). In addition, one Chinese material labeled as *R. cyanoxantha* (Schaeff.) Fr. is affiliated with European collections identified as *R. cyanoxantha* or *R. langei* Bon (Figure 1). The populations of the same species of subg. *Heterophyllidiae* between the two regions will be defined in the future.

The affinities of subg. *Heterophyllidiae* species between China and Southeast/South Asia are evident. For example, *R. lakhanpalii* A. Ghosh, K. Das, and R.P. Bhatt occurs in both China and India, and our new species *R. niveopicta* was shared between China and Thailand (Figure 1). Moreover, we also noted that *R. xanthovirens* and *R. subatropurpurea* are distributed in both China and Japan (Figure 1).

### Key to sections (subsection) of *Russula* subgen. *Heterophyllidiae* from China

The recognition of several sections in this subgenus for which already available names include *Ingratae*, *Heterophyllae*, and *Virescentinae*. Probably subsect. *Cyanoxanthinae* and *Substriatinae* also merit upgrading (Buyck et al., 2018).

**Table:** 

1. Pileus bright pink to green tones, pileipellis always metachromatic in Cresyl blue	subsect. *Cyanoxanthinae*
1. Pileus usually dull brown, white, or red tones, pileipellis orthochromatic in Cresyl Blue	2
2. Pileus often white, brown, or red tones, with distinct tuberculate-striate margin	3
2. Pileus often green to cinnamon tones, not striate or with inconspicuous striate	sect. *Heterophyllae*
3. Odor mostly mild, rarely acrid, pileipellis usually with short, inflated subterminal cells	4
3. Odor mostly distinct fetid, pileipellis usually with cylindrical, uninflated subterminal cells	sect. *Ingratae*
4. Pileipellis with aggregate, fusiform pileocystidia	subsect. *Substriatinae*
4. Pileipellis with segregate, clavate to subcylindrical pileocystidia	sect. *Virescentinae*

### Key to accepted species of *Russula* subsect. *Cyanoxanthinae* from China

**Table:** 

1. Pileus surface pale pink, grayish-pink, pale pinkish purple, lavender blush to rosy brown	2
1. Pileus surface pale ochre, olive green, dark green, green white to grayish green	5
2. Hymenophore without lamellulae, pileus margin crenate	*R. fusiformata*
2. Hymenophore with lamellulae, pileus margin even or incurved	3
3. Pileus center yellowish white, basidiospores ornamentation higher (up to 2 μm)	*R. lotus*
3. Pileus center rosy brown, pale pink or pale grayish-pink, basidiospores ornamentation lower (up to 0.7 μm)	4
4. Lamellae not forking, unchanging in color when injured, stipe cylindrical, cystidia negative in SV	*R. purpureorosea*
4. Lamellae often forking, sometimes becoming yellowish brown when injured, stipe slightly expanded toward the base, cystidia gray in SV	*R. subpallidirosea*
5. Pileal surface green, non-striate, stipe slightly attenuate toward the base, basidiospores 6.5–8.5 × 6–8 μm, ornamentation up to 0.6 μm	*R. nigrovirens*
5. Pileal surface pale ochre when young, then becoming olive green to dark green, mixed with the rusty tone, slightly striate with age, stipe cylindrical, basidiospores 6–8 × 5–7 μm, ornamentation up to 0.4 μm	*R. dinghuensis*

### Key to accepted species of *Russula* sect. *Virescentinae* from China

**Table:** 

1. Pileus not peeling readily	2
1. Pileus with readily peeling skin	4
2. Pleurocystidia negative in SV	*R. albidogrisea*
2. Pleurocystidia positive in SV	3
3. Basidiospores larger measuring 6.5–7.5 × 5.0–6 μm, ornamentation lower (0.2–0.45 μm), pleurocystidia becoming dark gray in SV	*R. pallidula*
3. Basidiospores smaller measuring 5–7 × 4.5–6 μm, ornamentation higher (0.4–0.7 μm), pleurocystidia becoming yellowish brown in SV	*R. niveopicta*
4. Appressed patched scales on the pileal surface	5
4. Pileus without patched scales	6
5. Pileal surface pinkish red or light jasper red on the margin and yellowish olive in the center, basidiospores ornamentation higher (0.6–1.2 μm)	*R. viridirubrolimbata*
5. Pileal surface purplish gray to grayish magenta toward the margin and grayish yellow to brownish orange in the center, basidiospores ornamentation lower (0.3–0.6 μm)	*R. luofuensis*
6. Pleurocystidia positive in SV	7
6. Pleurocystidia negative in SV	8
7. Pileal surface yellowish white to pinkish to dark pink, peeling to one-fourth of the radius, basidiospores ornamentation higher (0.4–0.8 μm), pleurocystidia becoming tawny in SV	*R. subpunicea*
7. Pileal surface yellowish white in the center, margin white, peeling to one-third of the radius, basidiospores ornamentation lower (0.3–0.5 μm), pleurocystidia becoming mauve in SV	*R. albolutea*
8. Pileal surface yellowish green to deep green, stipe white tinged with green, basidiospores ornamentation higher (0.4–0.8 μm)	*R. xanthovirens*
8. Pileal surface yellowish green to golden green, stipe white to pale cream, basidiospores ornamentation lower (up to 0.2 μm)	*R. aureoviridis*

### Key to accepted species of *Russula* sect. *Ingratae* from China

**Table:** 

1. Pileus with appressed patched scales	2
1. Pileus without patched scales	5
2. Basidiospores ornamentation higher (≥2.5 μm), composed of large wings	3
2. Basidiospores ornamentation lower (<2.5 μm), composed of ridges	4
3. Lamellulae absent, odor faint and fragrant, pleurocystidia blackening in SV	*R. subpunctipes*
3. Lamellulae rare, odor indistinct, pleurocystidia becoming reddish brown in SV	*R. gelatinosa*
4. Odor strongly fetid, basidiospores larger measuring 8–9.5 × 7.3–8.8 μm, pleurocystidia becoming blue in SV	*R. senecis*
4. Odor not distinctive, basidiospores smaller measuring 5.5–7 × 5–6.5 μm, pleurocystidia becoming brownish black in SV	*R. hainanensis*
5. Basidiospores ornamentation higher (>1.2 μm)	6
5. Basidiospores ornamentation lower (≤1.2 μm)	9
6. Odor distinct, basidiospores ornamentation forming an incomplete reticulum	7
6. Odor indistinct, basidiospores ornamentation never forming a reticulum	8
7. Odor strongly fetid, ornamentation composed of high wings (up to 3 μm)	*R. punctipes*
7. Odor intense frangipani, ornamentation composed of high ridges (up to 2 μm)	*R. guangdongensis*
8. Basidiomata larger (7–9.2 cm), pileus not peeling readily, hymenial cystidia turning blackish-gray in SV	*R. clavulus*
8. Basidiomata smaller (5–7.5 cm), peeling readily, hymenial cystidia turning yellowish brown in SV	*R. multilamellula*
9. Basidiospores ornamentation never forming a reticulum	10
9. Basidiospores ornamentation forming a complete or incomplete reticulum	11
10. Basidiospores smaller measuring 5.3–6.8 × 5–5.9 μm, hymenial cystidia grayish in SV	*R. indocatillus*
10. Basidiospores larger measuring 7–8.6 × 5.5–6.6 μm, hymenial cystidia negative in SV	*R. pseudocatillus*
11. Pileal surface dry, stipe often tinged with reddish brown, base reddish	*R. rufobasalis*
11. Pileal surface slightly viscous, stipe cream, white, pale yellowish brown or yellowish gray, base without reddish tinge	12
12. Context white, unchanging in color when injured, pleurocystidia blackish-gray in SV	*R. succinea*
12. Context slowly changing brown in color when injured, pleurocystidia grayish in SV	13
13. Basidiospores ornamentation higher (≥0.7 μm)	*R. straminella*
13. Basidiospores ornamentation lower (<0.7 μm)	14
14. Lamellae sometimes forked near the stipe, basidiospores smaller measuring 5.6–7 × 4.6–6 μm, suprahilar spot inamyloid and indistinct, a distribution in subtropical China	*R. subpectinatoides*
14. Lamellae rarely forked around the stipe, basidiospores larger measuring 6.5–9 × 5–7.5 μm, suprahilar area amyloid and distinct, a distribution in temperate China	*R. pseudopectinatoides*

### Key to accepted species of *Russula* sect. *Heterophyllae* from China

**Table:** 

1. Pileus margin with striate	2
1. Pileus margin without striate	3
2. Lamellae forking, basidia narrower (up to 12.9 μm)	5
2. Lamellae not forking, basidia wider (up to 15.6 μm)	*R. pseudobubalina*
3. Hymenophore with lamellulae, stipe usually tinged with pale greenish, cheilocystidia absent, a distribution in temperate China	*R. atroaeruginea*
3. Hymenophore without lamellulae, stipe white, cheilocystidia present, a distribution in subtropical or tropical China	4
4. Pileus purplish brown, not peeling readily, basidiospores ornamentation not forming a reticulum, hymenial cystidia becoming brown in SV	*R. subatropurpurea*
4. Pileus green tinged with cinnamon, peeling readily, basidiospores ornamentation forming an incomplete network, hymenial cystidia becoming dark gray in SV	*R. viridicinnamomea*
5. Basidiomata larger (pileus 5–10 cm in diameter), stipe white, cinnamon or blanched almond, basidiospores ornamentation forming an incomplete reticulum	6
5. Basidiomata smaller (pileus 3.5–5.4 cm in diameter), stipe light pink, basidiospores ornamentation not forming a reticulum	*R. bubalina*
6. Stipe white to cinnamon, basidiospores ornamentation higher (up to 0.7 μm), more pleurocystidia *ca.* 1,800/mm^2^, hymenial cystidia slightly becoming yellowish brown in SV	*R. discoidea*
6. Stipe white to blanched almond, basidiospores ornamentation lower (up to 0.5 μm), less pleurocystidia *ca.* 800–1,000/mm^2^, hymenial cystidia turning reddish black in SV	*R. subbubalina*

## Data availability statement

The datasets presented in this study can be found in online repositories. The names of the repository/repositories and accession number(s) can be found in the article/supplementary material.

## Author contributions

Z-QL and N-KZ contributed to the conceptualization, wrote, reviewed, and edited the manuscript, and supervised the data. Y-XH performed the methodology, wrote the original draft preparation, and carried out the formal analysis. N-KZ carried out the project administration and funding acquisition. All authors contributed to the article and approved the submitted version.

## Funding

This study was supported by the National Natural Science Foundation of China (No. 32160001), the Natural Science Foundation of Hainan Medical University (No. JBGS202112), and the Hainan Institute of National Park.

## Conflict of interest

The authors declare that the research was conducted in the absence of any commercial or financial relationships that could be construed as a potential conflict of interest.

## Publisher’s note

All claims expressed in this article are solely those of the authors and do not necessarily represent those of their affiliated organizations, or those of the publisher, the editors and the reviewers. Any product that may be evaluated in this article, or claim that may be made by its manufacturer, is not guaranteed or endorsed by the publisher.
